# Interactome of the Autoimmune Risk Protein ANKRD55

**DOI:** 10.3389/fimmu.2019.02067

**Published:** 2019-09-18

**Authors:** Nerea Ugidos, Jorge Mena, Sara Baquero, Iraide Alloza, Mikel Azkargorta, Felix Elortza, Koen Vandenbroeck

**Affiliations:** ^1^Neurogenomiks Group, Department of Neuroscience, University of the Basque Country (UPV/EHU), Leioa, Spain; ^2^Achucarro Basque Center for Neuroscience, Leioa, Spain; ^3^Proteomics Platform, CIC bioGUNE, CIBERehd, ProteoRed-ISCIII, Derio, Spain; ^4^IKERBASQUE, Basque Foundation for Science, Bilbao, Spain

**Keywords:** ANKRD55, ankyrin repeat, autoimmune, multiple sclerosis, rheumatoid arthritis

## Abstract

The ankyrin repeat domain-55 (*ANKRD55*) gene contains intronic single nucleotide polymorphisms (SNPs) associated with risk to contract multiple sclerosis, rheumatoid arthritis or other autoimmune disorders. Risk alleles of these SNPs are associated with higher levels of ANKRD55 in CD4^+^ T cells. The biological function of ANKRD55 is unknown, but given that ankyrin repeat domains constitute one of the most common protein-protein interaction platforms in nature, it is likely to function in complex with other proteins. Thus, identification of its protein interactomes may provide clues. We identified ANKRD55 interactomes via recombinant overexpression in HEK293 or HeLa cells and mass spectrometry. One hundred forty-eight specifically interacting proteins were found in total protein extracts and 22 in extracts of sucrose gradient-purified nuclei. Bioinformatic analysis suggested that the ANKRD55-protein partners from total protein extracts were related to nucleotide and ATP binding, enriched in nuclear transport terms and associated with cell cycle and RNA, lipid and amino acid metabolism. The enrichment analysis of the ANKRD55-protein partners from nuclear extracts is related to sumoylation, RNA binding, processes associated with cell cycle, RNA transport, nucleotide and ATP binding. The interaction between overexpressed ANKRD55 isoform 001 and endogenous RPS3, the cohesins SMC1A and SMC3, CLTC, PRKDC, VIM, β-tubulin isoforms, and 14-3-3 isoforms were validated by western blot, reverse immunoprecipitaton and/or confocal microscopy. We also identified three phosphorylation sites in ANKRD55, with S436 exhibiting the highest score as likely 14-3-3 binding phosphosite. Our study suggests that ANKRD55 may exert function(s) in the formation or architecture of multiple protein complexes, and is regulated by (de)phosphorylation reactions. Based on interactome and subcellular localization analysis, ANKRD55 is likely transported into the nucleus by the classical nuclear import pathway and is involved in mitosis, probably via effects associated with mitotic spindle dynamics.

## Introduction

An intronic single nucleotide polymorphism (SNP, rs6859219) in the ankyrin repeat domain protein-55 (*ANKRD55)* gene emerged originally as risk factor for rheumatoid arthritis (RA) from a meta-analysis of genome-wide association studies (1–3). We demonstrated that this SNP constitutes, as well, a genetic risk factor for multiple sclerosis (MS) (4), and subsequently we validated this association with genome-wide significance (5). A proxy of rs6859219 (*r*^2^ = 0.9, *D'* = 1), rs71624119, was also associated with MS in a comprehensive fine-mapping of autoimmune disease related genomic regions (6). Further studies have underscored pleiotropy of *ANKRD55* by linking SNPs in this locus to Crohn's Disease (7, 8), type 1 diabetes (9), juvenile idiopathic arthritis (10), celiac disease (11) and inflammatory myopathies (polymyositis and dermatomyositis) (12, 13), as well as post-traumatic stress disorder (14), cognitive decline in Alzheimer's disease (15), and type 2 diabetes (16, 17). Rs6859219 is significantly associated with expression levels of three *ANKRD55* transcripts, i.e., Ensembl (GRCh37.p13 Human Genome Assembly) full-length protein isoform 001, shorter protein isoform 005 and non-coding transcript 007 in PBMCs, and 001 and 005 in CD4^+^ T cells (18), and acts thus as a cis-expression quantitative trait locus (*cis*-eQTL). Rs71624119 (19) as wells as rs10065637 (20), a perfect proxy of rs6859219, were independently identified as *cis*-eQTLs for *ANKRD55* expression, the latter so by means of multiple tagging probes. The risk alleles of associated eQTL SNPs such as rs6859219, rs71624119 and rs10065637 are shared among autoimmune diseases and are associated with higher expression of ANKRD55 in CD4^+^ T lymphocytes (18–20). Thus, *ANKRD55* appears as the nearest annotated gene affected by the risk eQTL SNP rs6859219 or its proxies that emerged from GWAS on various autoimmune diseases. Notwithstanding these strong indications for a role of *ANKRD55* gene products in autoimmune pathogenesis, the biological function of ANKRD55 remains unclarified. Bioinformatic inspection of ANKRD55 does not provide clues as to its function beyond the presence of nine ankyrin repeats (ARs) in isoform 001, of which only the last is shared with isoform 005. ARs are 30–34 residue sequence motifs that represent one of the most common, modular, protein–protein interaction motifs in nature (21). They are found in proteins with diverse functions involved in processes such as transcription, cell cycle regulation, cytoskeletal integrity, inflammatory response, development, cell–cell signaling, and various transport phenomena (22, 23). The involvement of ankyrin repeat proteins in targeting, correct orientation and mechanical stabilization of membrane proteins to specific compartments within the endoplasmic reticulum (ER) and plasma membrane is well-documented (24). Various biologically important proteins including the family of INK4 tumor suppressors, p15, p16, p18, and p19, the signaling protein Notch and the NF-κB inhibitor IκB, as well as 53BP2, a regulator of the tumor suppressor p53, all contain ankyrin repeats (22). Ankyrin repeat motifs comprise typically a β-turn and two anti-parallel α-helices. In elucidated complexes, the β-turn and the first α-helix mediate the interactions with the target, and varying numbers of neighboring repeats partake in binding (25–27). These observations support a role for the ankyrin repeat as a versatile scaffold for protein–protein interactions (PPIs) (21), with reported binding affinities of natural ankyrin repeat proteins for their partners occurring in the low nanomolecular range (28). Based on its ankyrin repeats, ANKRD55 is thus likely to take part in interactions with diverse proteins. In this study we used a proteomics-based interactome approach in order to identify partners of ANKRD55 in purified complexes. Using this approach, protein partners whose individual roles in the cell have already been documented, were identified, and these thus shed light on the pathways and processes in which ANKRD55 is active.

## Materials and Methods

### Cell Culture

HEK293 and HeLa cell lines were maintained in Dulbecco's Modified Eagle's medium (DMEM) supplemented with 10% inactivated fetal bovine serum (FBSi) and 2 mM L-glutamine (all from Sigma-Aldrich).

### DNA Constructs and Cloning Procedures

pCMV6 containing TrueORF Gold Expression validated cDNA clone including the C-terminal DDK-myc tagged ORF of human ANKRD55 transcript 001 (NM_024669; Cat. No. RC221211) was purchased from OriGene. Human ANKRD55 transcript 005 coding sequence was amplified by PCR from pCR-BluntII-TOPO clone IRCMp5012E115D (Source Bioscience) with specific primers including restriction sites from Integrated DNA Technologies (IDT) using *Pfu* DNA Polymerase (Agilent Technologies) following manufacturer's protocol. Primers used: ATTCGCGATCGCCATGGACAGCAACCTG (005 *SgfI* Fw), ACCACGCGTATTTTCATCACTGGTGGGGTTGGCAGA (005 *MluI* Rv). The cycling conditions were set to initial denaturation at 94°C 45 s, followed by 30 cycles of denaturation at 94°C 45 s, annealing at 50°C 45 s and extension at 72°C 1 min 30 s and a final extension at 72°C for 10 min. The PCR product size was verified by agarose gel electrophoresis, and the products were purified using GeneJET Gel Extraction and DNA Cleanup Micro Kit (Thermo Fisher Scientific) following manufacturer's protocol. Amplified product was digested using *SgfI* and *MluI* restriction enzymes (New England Biolabs) and verified by agarose gel electrophoresis. Purified insert was ligated with pCMV6 vector digested with the same enzymes using T4 ligase (New England Biolabs). Individual colonies were screened by colony PCR and double digestion to identify those that contain the insert and confirmed by Sanger sequencing.

### ANKRD55 Expression Analysis and Immunoprecipitation

HEK293 and HeLa cells were cultured in T75 flasks (Sigma-Aldrich) and transfected with FLAG/myc ANKRD55 isoforms 001 or 005 using MACsfectin Reagent (Miltenyi Biotec) according to manufacturer's protocol and incubated for 48 h. Wherever applicable, cells were cross-linked using the membrane-permeable thiol-cleavable homobifunctional cross-linker dithiobis[succinimidyl propionate] (DSP;Thermo Fisher) dissolved in dimethyl sulfoxide. The cells were incubated in PBS supplemented with 100 μg/ml of DSP for 30 min, with gentle vortexing every 5 min. Reactions were quenched by adding 1 M Tris-HCl pH 7.5 to final concentration of 50 mM 15 min at room temperature (RT). For analysis of recombinant ANKRD55 isoform expression, HeLa and HEK293 were fractionated into different subcellular compartments following the protocol from Horton and Holden (29). FLAG-ANKRD55 immunoprecipitation (IP) was carried out from nuclear extracts obtained by sucrose gradient centrifugation (30); membranous organelles from subcellular fractionation and total protein extracts using lysis buffer (50 mM NaH_2_PO_4_, 300 mM NaCl, 1% triton X-100 and 1% cOmplete EDTA-free protease inhibitor cocktail pH adjusted to 8.0) or RIPA buffer (50 mM Tris-HCl, pH 7.5, 150 mM NaCl, 1% NP-40, 0.5% sodium deoxycholate and 0.1% SDS, 1 U/ml Benzonase and 1% cOmplete EDTA-free protease inhibitor cocktail). Part of the lysates were kept as input controls. For full interactome analysis, ANKRD55 IP was done from nuclear extracts lysed with RIPA buffer and total protein extracts using lysis buffer. Both buffers included phosphatase inhibitors (1 mM sodium pervanadate, 5 mM beta-glycerophosphate and 5 mM NaF). Part of the lysates were kept as input controls. Protein concentration was estimated using BCA protein assay kit (Pierce). For nuclear enrichment by sucrose gradient centrifugation, cells were washed three times with PBS and resuspended in ice-cold sucrose buffer I (0.32 M sucrose, 3 mM CaCl_2_, 2 mM magnesium acetate, 0.1 mM EDTA, 10 mM Tris-HCl pH 8.0, 1 mM DTT, 0.5% NP-40) in a 50-ml tube. A small aliquot of cells was examined with a phase-contrast microscope to ensure that they were uniformly lysed. Sucrose buffer II (2 M sucrose, 5 mM magnesium acetate, 0.1 mM EDTA, 10 mM Tris-HCl pH 8.0, 1 mM DTT) was added to 50 ml polypropylene centrifuge tubes for JA-20 rotor (sucrose cushion). Nuclei mixture were carefully layered onto the sucrose cushion, the gradient was completed with sucrose buffer I to have the same volume and centrifuged for 45 min at 30,000× g and 4°C in Beckman Coulter J2-MC High Speed Centrifuge. The supernatant was carefully removed, and nuclei were lysed with RIPA buffer. For the IP of ANKRD55 isoforms together with their binding partners, ANKRD55 and control samples were incubated with Anti-DDK G1 mouse monoclonal antibody coupled to the resin (cat.no. L00432; Genscript) at 4°C overnight (o/n), on rotating wheel. After incubation, resin was washed six times with washing buffer containing 50 mM Tris-HCl and 150 mM NaCl, pH adjusted to 7.4 (phosphatase inhibitors were included for interactome analysis) and ANKRD55 001 complexes were eluted using acidic elution buffer (0.1 M glycine, 0.15 M NaCl and 0,5% SDS pH 2).

### Interactome Analysis

#### In-gel Tryptic Digestion

Immunoprecipitated samples were separated by SDS-PAGE on 6% or 10% gels and stained with Pierce Silver Staining kit or SYPRO Ruby Protein Gel Stain (both from Thermo Fisher) according to manufacturer's protocol. Either selected protein bands from silver stained gels were excised, or entire lanes from SYPRO Ruby-stained gels were cut into ten contiguous pieces (done for both the ANKRD55 001 and control lanes). These gel slabs were then cut into small pieces and washed in Milli-Q water. Reduction and alkylation was achieved by incubation with dithiothreitol (DTT, 10 mM in 50 mM ammonium bicarbonate) at 56°C for 20 min, followed by incubation in iodoacetamide (IA, 50 mM in 50 mM ammonium bicarbonate) for another 20 min, in the dark. Gel pieces were dried and incubated with trypsin (12.5 μg/mL, in 50 mM ammonium bicarbonate) for 20 min on ice. After rehydration, the trypsin supernatant was discarded; gel bands were covered with 50 mM ammonium bicarbonate and incubated overnight at 37°C. After digestion, supernatant with digested peptides was recovered. Acidic peptides were further extracted from the gel with TFA 0.1% and pooled with the first supernatant. Digested peptides were dried in a RVC2 25 SpeedVac concentrator (Christ). Peptides were resuspended in 0.1% FA and sonicated for 5 min prior to their nano LC MS/MS analysis.

#### NanoLC-MS/MS and Data Analysis

Peptide mixtures obtained from trypsin digestion were separated by online nanoLC and analyzed by electrospray tandem mass spectrometry. Peptide separation was performed on a nanoAcquity UPLC system (Waters) connected to an LTQ Orbitrap XL mass spectrometer (Thermo Electron). Approximately 40% of each sample was loaded onto a Symmetry 300 C18 UPLC Trap column, 180 μm × 20 mm, 5 μm (Waters). The precolumn was connected to a BEH130 C18 column, 75 μm × 200 mm, 1.7 μm (Waters) equilibrated in 3% acetonitrile and 0.1% FA, and peptides were eluted at 300 nl/min using a 60 min linear gradient of 3–50% acetonitrile directly onto the nanoelectrospray ion source (Proxeon Biosystems). The mass spectrometer automatically switched between MS and MS/MS acquisition in DDA mode. Survey full scan MS spectra (m/z 400–2,000) were acquired in the orbitrap with a resolution of 30,000 at m/z 400. The 6 most intense ions were sequentially subjected to collision-induced dissociation (CID) fragmentation in the linear ion trap. Precursors with charge states of 2 and 3 were specifically selected for CID. Collision-energy applied to each peptide was automatically normalized as a function of the m/z and charge state. Analyzed peptides were excluded for further analysis during 30 s using dynamic exclusion lists. Searches were performed using Mascot Search engine (Matrix Science) on Proteome Discoverer 1.2. software (Thermo Electron). Carbamidomethylation of cysteines as fixed modification, oxidation of methionines as variable modification, 10 ppm of peptide mass tolerance, and 0.5 Da fragment mass tolerance were adopted as search parameters. Two missed cleavages were allowed. Spectra were searched against a UNIPROT/Swissprot database restricted to *Homo sapiens*. A false discovery rate estimation procedure was applied for peptide identification (FDR <1%). Proteins identified with at least two peptides passing that cutoff were considered for further discussion. For interactome analysis, three replicates were run for each sample type, and a spectral counting approach was carried out in order to select specifically enriched proteins. This approach relies on the counting of the MS/MS spectra matching to a certain protein (31). The comparison of this value for a certain protein in two different samples provides an idea of its putative differential abundance. In our case, proteins with an ANKRD55 IP/Negative Ctrl IP spectral count ratio >5 in 3/3 replicates were selected for further analysis and discussion. In order to determine the relative abundance of each protein in each of the samples, a Normalized Spectral Abundance Factor (NSAF) approach was carried out (32). The spectral counts for a protein were normalized to its length and further expressed as a % of the total of normalized spectral counts present in that particular sample.

### Phosphopeptide Enrichment

To enrich for phosphorylated peptides, titanium dioxide chromatography was performed essentially as described by Rigbolt et al. (33) with some modifications. Briefly, an equal volume of Titansphere material (GL Sciences) was mixed with a buffer containing 80% ACN / 1% TFA / 0.6 M glycolic acid. The slurry was incubated for 20 min at RT with end-over-end rotation. Sample buffer was adjusted to a final concentration of 60% ACN / 1% TFA before incubation with the beads. Titansphere beads were added to the sample in a 0.6 mg TiO_2_ / 100 peptide μg proportion and mixed 20 min at RTwith end-over-end rotation. Finally, Titansphere material with bound phosphopeptides was washed three times with 150 μl 60% ACN / 1% TFA and transferred on top of a C8 disc (Empore) placed in 200 μL pipette-tip. Phopshopeptide samples were sequentially eluted using a buffer containing 5% NH_4_OH and a buffer containing 25% ACN / 10% NH_4_OH, dried in a RVC2 25 SpeedVac concentrator (Christ), and resuspended in 2% ACN / 0.3% TFA before nano LC MS/MS analysis.

### Functional and Pathway Analysis

The DAVID tool from the NIH (https://david.ncifcrf.gov/) (34) was used for Gene Ontology (GO) enrichment analysis. Results from the functional annotation clustering were used to select the interesting GO terms, and only results with *p* < 0.05 for the modified Fisher's test were considered. Ingenuity Pathway Analysis was used for more detailed characterization of the molecular events lying behind the differential protein patterns under analysis. The calculated *p*-values determine the probability that the association between proteins in the dataset and a given canonical pathway or upstream regulator is explained by chance alone, based on a Fisher's exact test (*p*-value < 0.05 considered significant).

### Validation of ANKRD55 Partners

#### Validation by Reverse IP

HEK293 cells were transfected with the ANKRD55 001 vector construct using MACSfectin Reagent (Milteny Biotec), or left untransfected. Twenty-four hours later cells were collected and lysed in RIPA buffer (50 mM Tris-HCL, pH 7.5, 150 mM NaCl, 1% NP-40, 0.5% sodium deoxycholate, 0.1% SDS and 1X cOmplete EDTA-free protease inhibitor cocktail) for 30 min on ice. 0.1 ml of cell lysates were immunoprecipitated overnight in a rotating wheel at 4°C with 3 μg of anti-14-3-3 epsilon rabbit polyclonal (Cat. No. 11648-2AP), 3 μg of anti-RPS3 rabbit polyclonal (Cat. No. 11990-1-AP), 3 μg of IgG control rabbit polyclonal (Cat. No. 30000-0-AP), 5 μg of anti-TUBB antibody mouse monoclonal (Cat. No. 66240-1-Ig) or 5 μg of IgG control mouse polyclonal (Cat. No. B900620), all from Proteintech. Next, 20 μl of protein A resin (Cat. No. L00400, GeneScript) was added and incubation was continued in a rotating wheel for 2 h at 4°C. After incubation, protein A resin was washed 3 times with TBS-T (0.2%) and IP were eluted in 20 μl of 2 × SDS loading solution, resolved on SDS-PAGE and transferred to PVDF membranes. ANKRD55 001 was detected in the IPs with anti-ANKRD55 (Cat. No. HPA051049; 1:500; from Sigma-Aldrich). 14-3-3 with anti-14-3-3 (pan) (Cat. No. 8312; 1:1,000; from Cell Signaling). RPS3 with anti-RPS3 (Cat. No. 11990-1-AP; 1:2,000; from Proteintech), and TUBB with anti-TUBB (Cat. No. 66240-1-Ig; 1:1,000; Proteintech). HRP couple protein A (Cat. No. SA00001-18; 1:6,000) from Proteintech was used to detect primary antibodies.

#### Validation by Immunocytochemistry

For colocalization studies of endogenous and recombinant ANKRD55 with interacting partners, HEK293 cells were plated on coverslips coated with poly-D-lysine (Sigma-Aldrich) and transfected with ANKRD55 001 using MACsfectin Reagent (Miltenyi Biotec) according to manufacturer's instructions. 24 and 48 h later, cells were fixed with 4% paraformaldehyde in PBS for 20 min at RT. Those coverslips destined to nuclear colocalization were subjected to antigen retrieval treatment by incubating the cells with R-Universal buffer (Cat. No. AP0501-500; Aptum). Next cells were permeabilized with 0.2% Triton X-100 in PBS for 30 min and blocked with 3% BSA (all from Sigma-Aldrich) in PBS for 30 min. Staining was performed with anti-ANKRD55 rabbit polyclonal (1:100), anti-RPS3 mouse monoclonal (Cat. No. 66046-1-Ig; 1:500), anti-TUBB mouse monoclonal (1:200), anti-VIM mouse monoclonal (Cat. No. 60330-1-Ig; 1:200), anti-14-3-3 mouse monoclonal (Cat. No. 66061-1-Ig; 1:50), and/or anti-FLAG rabbit polyclonal (Cat. No. 20543-1-AP; 1:200), all from Proteintech for 1 h at RT, followed by staining with Alexa Fluor 647 conjugated anti-rabbit (ab150075; 1:500) and DyLight 550 conjugated anti-mouse (ab98713; 1:500) from Abcam for 1 h at RT and counter-staining with DAPI (1:500; Sigma-Aldrich). Images were obtained using a Leica TCS CW SP8 STED Super-Resolution microscope with a 63x immersion objective and excitation wavelengths of 405, 551, and 650 nm.

#### Quantification of Colocalization

For the cytoplasmatic colocalization, quantification was carried out using LAS AF (Leica Application Suite Advanced Fluorescence) software and Pearson's correlation coefficient. First, the background signal was extracted in all the studied images. Then, the Pearson's correlation coefficient values from 10 regions of interest (ROIs) were annotated, corresponding each ROI to a unique cell (*n* = 10 cellular ROIs/condition). In transfected cells the ROIs were selected from transfected cells. Analysis of the colocalization quantification was performed with GraphPad v.6 (GraphPad Software, San Diego, CA, USA). The data is represented as mean ± SEM and the Mann–Whitney test was applied. The level of significance was set at *p* ≤ 0.05, with *p* ≤ 0.0001 (^****^) as extremely significant, *p* ≤ 0.001 (^***^) highly significant, *p* ≤ 0.01 (^**^) very significant, *p* ≤ 0.05 (^*^) significant, and *p* > 0.05 (ns) not significant.

#### Validation by Western Blot

**Figure 1:**
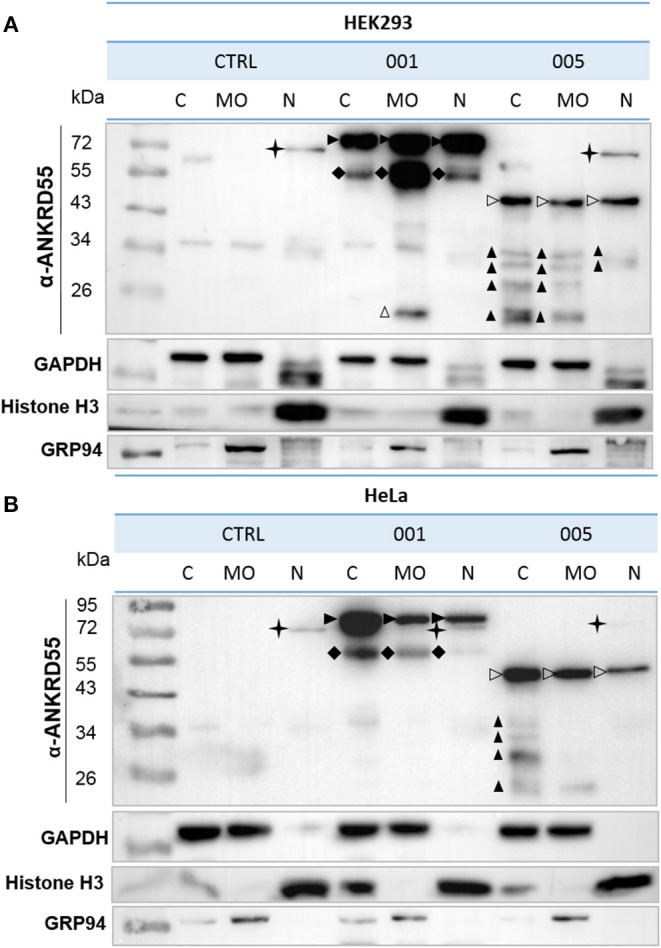
Subcellular localization of recombinant ANKRD55 isoforms in HEK293 and HeLa cells. **(A)** HEK293 and **(B)** HeLa cells were transfected with vectors expressing either isoform 001 or 005 of ANKRD55 or left untransfected (negative control). Cells were fractionated at 48 h after transfection. ANKRD55 was detected with specific Ab (Sigma-Aldrich) by WB. Enrichment for subcellular fractions was verified using histone H3 as nuclear marker (N), GRP94 as marker of membranous organelles (MO) and GAPDH as cytosolic marker (C). Specific bands corresponding to overexpressed isoforms 001 and 005 are indicated by black and white arrowheads, respectively. Black cross marks endogenous ANKRD55 isoform 001. Black diamonds, and black and white triangles show three different types of truncated immunoreactive products.

ANKRD55 isoforms in immunoblots were detected by anti-ANKRD55 rabbit polyclonal (1:500), anti-FLAG rabbit polyclonal (1:1,000), anti-RPS3 mouse monoclonal (1:2,000), anti-CLTC mouse monoclonal (Cat. No. 66487-1-Ig; 1:1,000), anti-VIM mouse monoclonal (1:1,000), anti-TUBB mouse monoclonal (1:5,000) and anti-SMC1A rabbit polyclonal (Cat. No. 21695-1-AP; 1:500) from Proteintech; anti-SMC3 rabbit monoclonal (Cat. No. 5696; 1:2,000), anti-14-3-3 pan (1:1,000) from Cell Signaling Technology; anti-PRKDC mouse monoclonal (Cat. No. NBP2-22128SS; 1:1,000) and anti-HIF1AN rabbit polyclonal (Cat. No. NB100-428; 1:1,000) from Novus Biologicals and subsequently with HRP-conjugated anti-rabbit (Cat. No. 111-035-144; 1:5,000) or anti-mouse (Cat. No. 715-035-150; 1:3,000) from Jackson Immunoresearch and anti-rat (Cat. No. 7077; 1:3,000) from Cell Signaling Technology secondary antibodies for 1 h at RT. The membranes were incubated with clarity Western ECL Substrate (Bio-Rad) for chemiluminescent signal detection using ChemiDoc camera (Bio-Rad). Anti-GAPDH mouse monoclonal (Cat. No. MAB374; 1:1,000) from Merck Millipore, anti-histone H3 rabbit polyclonal (Cat. No. 9715; 1:1,000) from Cell Signaling Technology, and anti-GRP94 rat monoclonal (Cat. No. ADI-SPA-850-D; 1:1,000) from Enzo Life Sciences Abs were used to validate cell fraction purity (Figure 1).

### Bioinformatics Analysis

ANKRD55 phosphosites were identified *in silico* using several tools: (i) PhosphoSitePlus (35) and dbPAF (36) databases were consulted to look for any ANKRD55 phosphosites described in scientific literature or public databases, and (ii) 14-3-3-Pred web server (37) was used to predict putative 14-3-3 binding phosphosites on ANKRD55 by combining predictions from three different classifiers: ANN—Artificial Neural Network (cut-off = 0.55), PSSM—Position-Specific Scoring Matrix (cut-off = 0.80), and SVM—Support Vector Machine (cut-off = 0.25). The ability of ANKRD55 for binding to nucleotides, DNA and RNA was analyzed using the following web-servers: (i) DRNApred (http://biomine.cs.vcu.edu/servers/DRNApred) (38) for the prediction of DNA and RNA-binding proteins and (ii) NsitePred (http://biomine.ece.ualberta.ca/nSITEpred/) (39) predicted binding residues from the protein sequence for ATP, ADP, AMP, GTP, and GDP.

## Results

### Overexpressed ANKRD55 Isoforms Are Located in Cytosol, Membranous Organelles and Nucleus

Human ANKRD55 001 and 005 isoforms were transiently overexpressed in HEK293 and HeLa cells, and cells were fractionated into three subcellular compartments; cytosol (C), nucleus (N) and membranous organelles (MO). In transfected HEK293 cells, similar levels of isoform 005 were detected in the three fractions. Isoform 001 (72 kDa) showed a more abundant signal than 005 (43 kDa), and was as well present in the three compartments though slightly less abundant in the cytosol than in the other two fractions (Figure 1). In HeLa cells, isoform 001-enriched fraction from the cytosol showed a stronger signal than that of the other two fractions. Isoform 005 detection was slightly weaker in nuclei than in the rest of the fractions. Moreover, truncated immunoreactive products were present. These included (i) a 55-60-kDa band in HEK293 and HeLa cells in isoform 001-transfected cells present in all 3 compartments though at levels differing from the full-length proteins, (ii) a 20-kDa band in MO of HEK293 cells, and (iii) a series of bands with Mr<34 kDa especially enriched in the cytosol of isoform-005 transfected HEK293 and HeLa cells. Additionally, the results showed the endogenous isoform 001 in both cell lines demonstrating the intrinsic capacity of HEK293 and Hela cells to produce ANKRD55 and therefore to engage its biological pathway(s). Thus, western blot analysis of biochemically fractionated cells revealed presence of the isoforms 001 and 005 of ANKRD55 in all three analyzed subcellular compartments of both cell lines (Figure 1). Further experiments showed that it was possible to efficiently purify ANKRD55 isoform 001, more so than isoform 005, using anti-FLAG agarose from MO fraction, nuclear (NE) and total (TPE) protein extracts in the presence or absence of cross-linker DSP (Supplementary Figure 1).

### Identification of Proteins in Bands Co-purifying With ANKRD55 Isoforms 001 and 005

FLAG ANKRD55 IP from MO fraction, TPE and NE were separated by SDS-PAGE and gels were silver stained. Numerous specific bands were visualized in FLAG-ANKRD55 001 and 005 IP that were not present in control samples (see examples in Supplementary Figure 2). Use of the cross-linker DSP had generally little effect on overall intensities of the bands, suggesting that the interaction with ANKRD55 was unaffected by lysis and purification conditions. Bands corresponding to the Mr of recombinant FLAG-ANKRD55 isoforms and those not present in the control samples were cut, trypsin digested and identified by nLC-MS/MS analysis (Supplementary Table 1). Proteins belonging to the 14-3-3 family (YWHAE, YWHAZ, YWHAH, YWHAB, YWHAQ, YWHAG) were repeatedly identified in IPs of ANKRD55 001 or 005 from TPE but not those from NE or MO extracts. Heat shock proteins (HSPA9, HSPA5, HSPA1A, HSP90B1, HSP90AB1, HSP90AA1, HSPA8, HSPD1) were also abundant among ANKRD55 interactors especially so in the MO fraction. HSP90AB1, HSP90AA1 and the unique HSP90 ER homolog GRP94 (HSP90B1) were identified in ANKRD55 IPs from MO fractions of both HEK293 and HeLa cells. Members of the tubulin superfamily were found in ANKRD55 001 IPs from TPE and NE but not MO (TUBB, TUBA1B, TUBB4B, TUBB4A). Proteins participating in initiation and elongation phases of eukaryotic translation (EEF1A1, EIF4A1, TUFM, EIF4A3, EEF1G, EEF2) were identified in all extracts. Members of the mitochondrial transporter family SLC25 (SLC25A1, SLC25A5, SLC25A6) were identified in TPE and MO extracts. YWHAE was identified in presence or absence of cross-linker as well as in two independent experiments from HEK293 TPE. The protein kinase PRKDC was found in three independent experiments in HEK293 NE. Thus, this first analysis revealed that ANKRD55 interacts (in)directly with a combination of functionally distinct proteins in MO, NE and TPE. Of these, 14-3-3 proteins appear to present the best-resolved interactors in silver-stained gels (various examples in Supplementary Figure 2).

### The ANKRD55 Interactome

**Figure 2:**
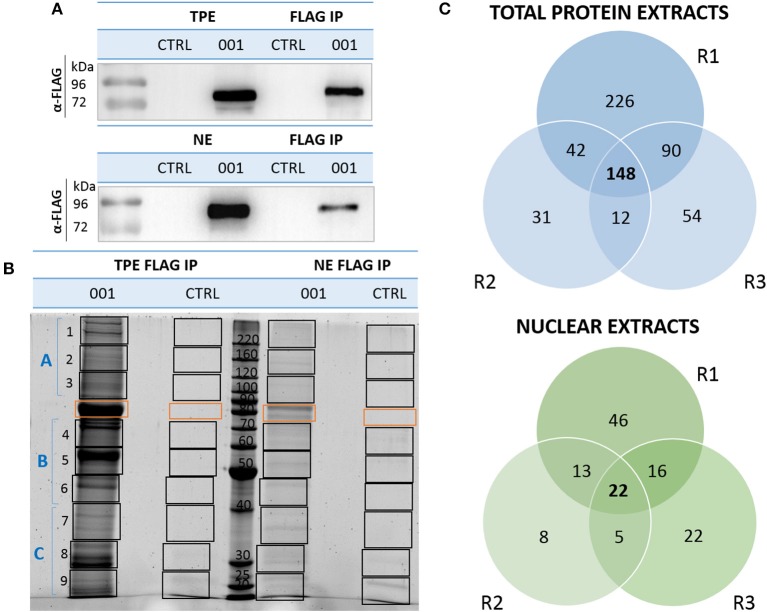
Isolation and identification of ANKRD55 interactome components by nLC-MS/MS. **(A)** Recombinant ANKRD55 isoform (001) was detected in nuclear extracts (NE), total protein extracts (TPE) and eluted fractions (FLAG IP) with FLAG Ab by WB. As negative control, cells were cultivated under the same conditions, but without transfection agent (CTRL). **(B)** A SYPRO Ruby-stained SDS-PAGE showing analysis strategy of ANKRD55 complexes IP with FLAG resin from NE and TPE of HEK293 cells expressing isoform 001. ANKRD55 band or corresponding position (CTLR) is indicated with orange rectangle, while black rectangles show contiguous gel slabs processed for mass spec. WB and SDS-PAGE are representative of three independent biological replicates. **(C)** Venn diagram shows numbers of unique and shared interactors in each independent replicate (R1, R2, and R3) out of three performed for ANKRD55 IP from TPE and NE.

We compared and analyzed in more detail ANKRD55 and control IPs in order to search for and identify additional interacting partners as well as to confirm those identified above. In essence, rather than focusing on single visualized gel bands, we analyzed the complete gel lanes corresponding to ANKRD55 001 or mock (not transfected cells) FLAG eluates. Low abundant ANKRD55-interacting proteins may be present that are not resolved as single bands, and therefore could be missed by the first method. Together such bands tend to produce a darker stainable protein smear in ANKRD55 compared to mock lanes (several such examples can be seen in Supplementary Figure 2). By subtracting the “mock proteome” from the ANKRD55 proteome, a specific and comprehensive ANKRD55 interactome can be deduced. We focused this proteomic analysis on FLAG-tagged ANKRD55 isoform 001, which was more efficiently IPed than isoform 005, and showed highest number of potential interacting partners. FLAG-ANKRD55 isoform 001 was overexpressed in HEK293 cells and IPed from TPE and sucrose gradient-purified NE in triplicates (three independent experiments each that were performed on distinct days from distinct batches of cells). WB with FLAG antibody was performed to verify the quality of ANKRD55 overexpression and IP (Figure 2A). ANKRD55 complexes were then separated by SDS-PAGE, and stained using SYPRO Ruby. The full gel lanes starting from the boundary of the stacking gel with the resolving gel up to the gel front were cut into ten pieces (one contained ANKRD55 001 or its control), trypsin digested, and identified by nLC-MS/MS (Figure 2B). To build the ANKRD55 interactome, all potential ANKRD55 interacting partners were filtered according to their spectral count enrichment. Spectral counting accounts for the total number of MS/MS spectra matching to peptides that belong to a certain protein, and provides a valuable parameter for relative protein abundance comparison (see section Materials and Methods for more details). We identified enrichment of 148 proteins in TPE and 22 proteins in NE over the three replicates, based on stringent selection criteria including both at least two unique peptides and a 5-fold increase in ANKRD55 spectral counts in the ANKRD55 IP compared to control IP in all three replicates. Several of these proteins were selected for further functional analysis and validation (Figure 2C, Table 1, Supplementary Table 2). The average NSAF of these proteins was calculated in order to estimate their relative abundance within the IPed samples. Although not considered for further analyses, an additional, less robust, dataset of putative ANKRD55 interactors was substracted in addition to the set of reliably enriched proteins. For this purpose, proteins enriched with a more relaxed criteria of ANKRD55 IP/negative control IP spectral count ratio of at least 5 in only 2 out of 3 replicates were selected, providing additional 144 putative-ANKRD55 partners in total protein extracts and 34 in nuclear extracts (Supplementary Table 3).

**Table 1:** Identification of the top twenty-two ANKRD55-interacting partners from total protein extracts and nuclear extracts from HEK293 cells, ranked by NSAF value.

	**Gene symbol**	**Accession**	**MW (kDa)**	**Protein**	**NSAF**	
					**ANKRD55**	**CTRL**
Total protein extracts	YWHAE	P62258	29.17	14-3-3 protein epsilon	2.64	0.21
	YWHAB	P31946	28.082	14-3-3 protein beta/alpha	1.14	0.00
	SLC25A5	P05141	32.852	ADP/ATP translocase 2	1.08	0.06
	YWHAH	Q04917	28.219	14-3-3 protein eta	0.97	0.00
	RPS3	P23396	26.688	40S ribosomal protein S3	0.81	0.26
	YWHAG	P61981	28.3	14-3-3 protein gamma	0.81	0.00
	TUBB6	Q9BUF5	49.857	Tubulin beta-6 chain	0.76	0.00
	SLC25A11	Q02978	34.062	Mitochondrial 2-oxoglutarate/malate carrier protein	0.66	0.08
	NTPCR	Q9BSD7	20.713	Cancer-related nucleoside-triphosphatase	0.57	0.00
	RPS15A	P62244	14.84	40S ribosomal protein S15a	0.53	0.00
	SLC25A13	Q9UJS0	74.176	Calcium-binding mitochondrial carrier protein Aralar2	0.47	0.00
	RPL9	P32969	21.863	60S ribosomal protein L9	0.46	0.00
	ARF4	P18085	20.511	ADP-ribosylation factor 4	0.46	0.00
	CCT4	P50991	57.924	T-complex protein 1 subunit delta	0.43	0.12
	PGAM5	Q96HS1	32.004	Serine/threonine-protein phosphatase PGAM5, mitochondrial	0.40	0.06
	MCM7	P33993	81.308	DNA replication licensing factor MCM7	0.39	0.00
	PCBP2	Q15366	38.58	Poly(rC)-binding protein 2	0.39	0.00
	PCBP1	Q15365	37.498	Poly(rC)-binding protein 1	0.37	0.00
	TIMM50	Q3ZCQ8	39.646	Mitochondrial import inner membrane translocase subunit TIM50	0.37	0.00
	RPL23	P62829	14.865	60S ribosomal protein L23	0.34	0.00
	PPP3CA	Q08209	58.688	Serine/threonine-protein phosphatase 2B catalytic subunit alpha isoform	0.34	0.00
	PCNA	P12004	28.769	Proliferating cell nuclear antigen	0.33	0.00
Nuclear extracts	VIM	P08670	53.65	Vimentin	1.11	0.19
	PHB2	Q99623	33.296	Prohibitin-2	1.03	0.08
	DDX3X	O00571	73.243	ATP-dependent RNA helicase DDX3X	0.81	0.11
	PGAM5	Q96HS1	32.004	Serine/threonine-protein phosphatase PGAM5, mitochondrial	0.81	0.00
	TUBA1C	Q9BQE3	49.895	Tubulin alpha-1C chain	0.73	0.00
	RPS3	P23396	26.688	40S ribosomal protein S3	0.64	0.00
	SMC1A	Q14683	143.23	Structural maintenance of chromosomes protein 1A	0.60	0.02
	DDX21	Q9NR30	87.344	Nucleolar RNA helicase 2	0.58	0.00
	ATAD3A	Q9NVI7	71.369	ATPase family AAA domain-containing protein 3A	0.57	0.04
	SMC3	Q9UQE7	141.54	Structural maintenance of chromosomes protein 3	0.49	0.02
	PRKDC	P78527	469.089	DNA-dependent protein kinase catalytic subunit	0.46	0.04
	NXF1	Q9UBU9	70.182	Nuclear RNA export factor 1	0.40	0.04
	RUVBL1	Q9Y265	50.22	RuvB-like 1	0.36	0.00
	RBM14	Q96PK6	69.492	RNA-binding protein 14	0.32	0.03
	AIFM1	O95831	66.901	Apoptosis-inducing factor 1, mitochondrial	0.30	0.00
	ELAVL1	Q15717	36.092	ELAV-like protein 1	0.29	0.00
	IRS4	O14654	133.768	Insulin receptor substrate 4	0.24	0.00
	WDR6	Q9NNW5	121.725	WD repeat-containing protein 6	0.22	0.00
	NUP205	Q92621	227.922	Nuclear pore complex protein Nup205	0.19	0.01
	NUP160	Q12769	162.121	Nuclear pore complex protein Nup160	0.16	0.00
	PDCD11	Q14690	208.701	Protein RRP5 homolog	0.09	0.00
	RANBP2	P49792	358.199	E3 SUMO-protein ligase RanBP2	0.09	0.00

*The rest of ANKRD55-interacting proteins identified from total protein extracts using nLC-MS/MS are listed in Supplementary Table 2*.

### Bioinformatic Analysis of ANKRD55 Protein Partners

**Figure 3:**
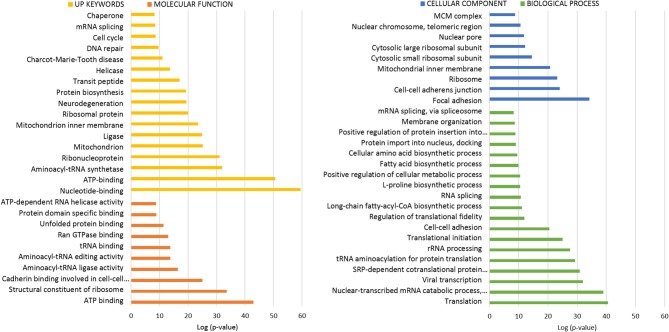
Functional enrichment analysis of ANKRD55 interactome from total protein extracts. The algorithm DAVID was used to analyze the ANKRD55 interactome using the GO terms Cellular Component (blue), Biological Process (green), Molecular Function (orange) and Up Keywords (yellow). The GO and other terms with FDR<5% are shown. Plotted as -log (*p*-value) significance.

**Figure 4:**
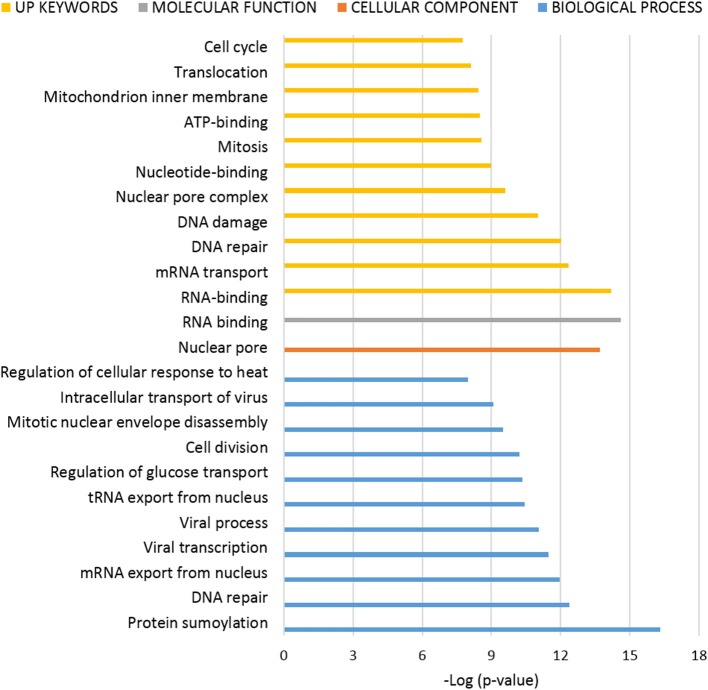
Functional enrichment analysis of ANKRD55 interactome from nuclear extracts. The algorithm DAVID was used to analyze the ANKRD55 interactome using the GO terms Cellular Component (orange), Biological Process (blue), Molecular Function (gray) and Up Keywords (yellow). The GO and other terms with FDR<5% are shown. Plotted as –log (*p*-value) significance.

To interpret the potential functional role of ANKRD55-interacting proteins, ANKRD55 interactome results were subjected to enrichment analysis using DAVID bioinformatics resource tool. We used functional annotation clustering to select the interesting Gene Ontology (GO) and other terms. The results from GO biological process, GO cellular compartment, GO molecular function and Uniprot (UP) keywords with a FDR<5% are shown in Figures 3, 4. A more detailed summary of the functional annotation clustering with *p* < 10^−3^ is provided in Supplementary Table 4. The functional analysis suggested that ANKRD55 protein partners from total protein extracts are tightly related to nucleotide and ATP binding (*p* < 1.18 × 10^−13^). Bioinformatics analysis predicted the binding ability of ANKRD55 to DNA, ATP, ADP and GTP nucleotides, but not to RNA (Supplementary Table 5). GO categories were enriched in nuclear transport terms including Ran GTPase binding, intracellular protein transport, protein import into nucleus and nuclear pore (*p* < 2.00 × 10^−03^). Total protein extracts dataset was also linked to processes that are associated with cell cycle and RNA, lipid and amino acid metabolism. Additionally, bioinformatics identified protein biosynthesis-related terms such as translation, structural constituent of ribosome, SRP-dependent cotranslational protein targeting to membrane, tRNA aminoacylation for protein translation and regulation of translational fidelity. GO cellular component annotated focal adhesion, ribosome, nuclear pore, and nuclear chromosome, among others, as the localization of the involved proteins in the total protein interactome. In the enrichment analysis of the ANKRD55-protein partners from nuclear extracts, ~20 and 30% of proteins are related to sumoylation and RNA binding, respectively. GO categories were involved in processes that are associated with cell cycle including cell division, mitotic nuclear envelope disassembly, mitosis, DNA repair and damage. Nuclear extracts interactome was also linked to processes including RNA transport, nucleotide and ATP binding, among others. GO cellular component analysis indicated nuclear pore as the localization of the involved proteins in the nuclear interactome. To further examine the ANKRD55 interactome, we performed an Ingenuity Pathway Analysis (IPA, QIAGEN) to identify major canonical pathways associated with ANKRD55-interacting proteins. Sixty out of one hundred ninety-six pathways were significantly enriched in total protein extracts (*p* < 0.05; –log (*p*-value) > 1.30). The top ten pathways, ranked by significance included tRNA Charging, EIF2 Signaling, Regulation of eIF4 and p70S6K Signaling, RAN Signaling, mTOR Signaling, Cell cycle: G2/M DNA Damage Checkpoint Regulation, HIPPO Signaling, Cell Cycle Control of Chromosomal Replication, PI3K/AKT Signaling and p70S6K Signaling. In nuclear extract interactome analysis, SMC1A and SMC3 proteins were related to Mitotic Roles of Polo-Like Kinase and ATM Signaling; PRKDC and PHB2 to Estrogen Receptor Signaling, VIM and TUBA1C to 14-3-3-mediated Signaling and PRKDC and TUBA1C to Sirtuin Signaling Pathway. All information on the IPA canonical pathways analysis of both interactomes is included in Supplementary Table 6.

### Validation of Selected ANKRD55 Interacting Partners by IP and Reverse IP

**Figure 5:**
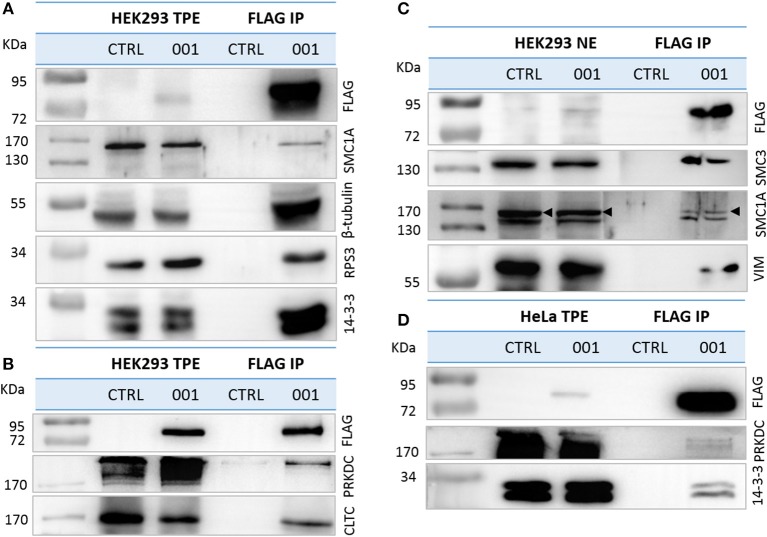
Validation of ANKRD55 isoform 001 interacting proteins. Overexpressed FLAG-ANKRD55 isoform 001 (001) associated with endogenous candidate proteins was IPed from **(A,B)** total protein extracts (TPE) and from **(C)** enriched nuclear fractions (NE), both obtained from HEK293 cells, as well as from **(D)** TPE of HeLa cells, after 48 h of transfection. As negative control cells were cultivated under the same conditions, but without transfection agent (CTRL). Recombinant ANKRD55 and endogenous proteins were detected by WB in NE, TPE and eluted fractions (FLAG IP) using FLAG Ab and antibodies specific for interacting partners identified by mass spec.

We confirmed the interaction of a selection of identified proteins with recombinant ANKRD55 isoform 001 by IP followed by WB using antibodies against the selected partners (Supplementary Table 7). From the cytosolic interactome, we selected (i) six of the first ten proteins ranked according to NSAF value (14-3-3 isoforms YWHAE, YWHAB, YWHAH and YWHAG, RPS3, and TUBB6); (ii) CLTC with a lower NSAF value which is mainly localized to coated vesicles and coated pits, in addition to cytosol and mitotic spindle; and (iii) proteins that were found to be present as well in the nuclear interactome (SMC1A and PRKDC). From the nuclear interactome, three proteins (VIM, SMC1A and SMC3) from the top ten in the list were analyzed. Several of these proteins had at first been identified in individual bands cut from silver-stained gels including PRKDC, SMC1A and SMC3 (see Supplementary Table 1). For detection of TUBB6, we used an anti-β-tubulin antibody capable of recognizing different β-tubulin isoforms including TUBB3, TUBB2B, TUBB, TUBB4B, TUBB2A, and TUBB4A (three of these were identified in two out of three interactome replicates, and several of these had been identified as well as in individual bands analyzed in the first approach, see Supplementary Table 1). Anti-FLAG IP experiments and WB demonstrated that endogenous SMC1A, TUBB6, RPS3, 14-3-3 isoforms, PRKDC, CLTC in total protein extracts (Figures 5A,B) and SMC1A, SMC3, VIM in nuclear extracts interacted specifically with ANKRD55 isoform 001 (Figure 5C). Neither ANKRD55 nor interacting partners were detected in the control IP. The IP of FLAG-ANKRD55 with 14-3-3 isoforms and PRKDC was also validated in total protein extracts of HeLa cells by WB (Figure 5D).

**Figure 6:**
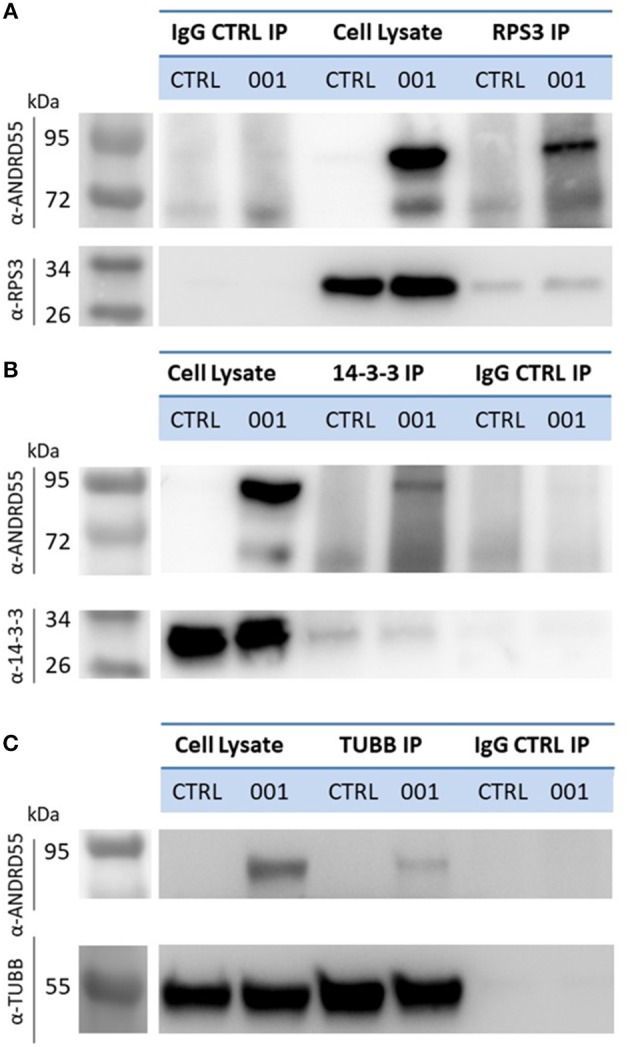
Detection of ANKRD55 in reverse immunoprecipitates of RPS3, 14-3-3ε and β-tubulin. RPS3 **(A)**, 14-3-3ε **(B)**, and β-tubulin **(C)** were immunoprecipitated from HEK293 transfected with ANKRD55 (001) vector or left untransfected (CTRL). Mock IPs were performed with control IgG antibodies as described in the Materials and Methods. IPs were resolved on SDS-PAGE, blotted and ANKRD55 was detected by means of anti-ANKRD55 Ab.

We performed also reverse IP using antibodies against 14-3-3 epsilon, the highest scoring partner of ANKRD55 in terms of NSAF value (Table 1), RPS3 and TUBB (Figure 6). Recombinant ANKRD55 was detected in the IPs of the specific antibodies but not in the IgG control IPs confirming that interactions of ANKRD55 with the target proteins were specific. We could not detect ANKRD55 in IPs from untransfected cells, likely due to the low levels of endogenous protein (Figure 1).

We checked freely available protein-protein databases (BioGRID (40), STRING (41) and HuRI^1^) to confirm some of the identified interactions. The BioPlex 2.0 dataset (42, 43), which has been deposited into the BioGRID database, described thirteen ANKRD55-interacting partners; seven were included in at least two out of three replicates from our interactome analysis (YWHAE, YWHAQ, IFT52, IFT74, MIB1, YWHAZ, and YWHAG). Intriguingly, HIF1AN, an ANKRD55-interacting partner described in the BioPlex 2.0 dataset, catalyzes hydroxylation of highly conserved asparaginyl residues within AR of NFKB1, NFKBIA, NOTCH1, ASB4, PPP1R12A and several other AR-containing proteins (44). Therefore, this could suggest that hydroxylation of asparaginyl residues from ANKRD55 may take place. However, in our experimental set-up, we did not clearly detect HIF1AN in ANKRD55 interactomes by mass spectrometry, and neither were we able to demonstrate clearly its interaction with ANKRD55 by WB analysis using specific antibodies (Supplementary Figure 3). STRING database also included ANKRD55-partners shared with our interactome, specifically RPS3 and ACLY in Homo sapiens and Rps3 and Hsp90ab1 in Mus Musculus. Human Reference Protein Interactome Project (HuRI) included thirty unpublished ANKRD55-interacting partners; none of these were classified in our protein list (Supplementary Table 8). However, some of the HuRI ANKRD55 partners are involved in ciliogenesis and its regulation (CFAP206, LRGUK, and DEUP1), just as are some proteins from our ANKRD55 interactome (MIB1, IFT74, ARF4, CCT4, CCT8, TTC26, FLNA, and IFT52), and ANKRD55 itself was described as a novel intraflagellar transport complex protein (45). Moreover, the BioGRID database also identified other members included in the same process (IFT46, TTC30A, and TTC30B).

### Immunofluorescence (IF) Colocalization of ANKRD55 and Partners

**Figure 7:**
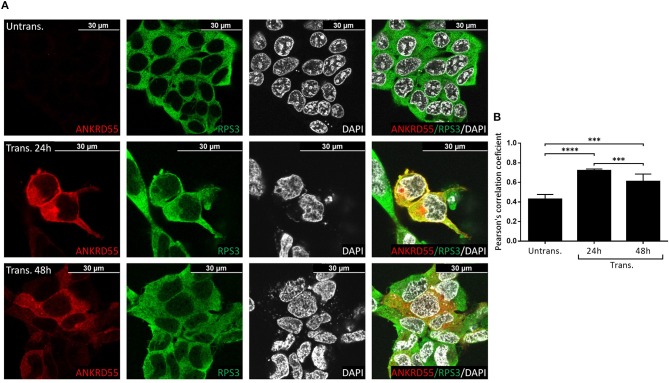
Recombinant ANKRD55 isoform 001 colocalizes with endogenous RPS3 in HEK293 cells. **(A)** Representative microphotographs of immunostaining for ANKRD55 (anti-ANKRD55 Ab; red), 40S ribosomal protein S3 (RPS3 Ab; green) and nuclei (DAPI; white) in untransfected (Untrans.) and transfected (Trans. 24h or 48h) HEK293 cells with ANKRD55 isoform 001. **(B)** Colocalization quantification using Pearson's correlation coefficient. Data are mean ± SEM (*n* = 10 cellular ROIs/condition), *p* ≤ 0.0001 (^****^) and *p* ≤ 0.001 (^***^) comparing three conditions, Mann-Whitney test.

**Figure 8:**
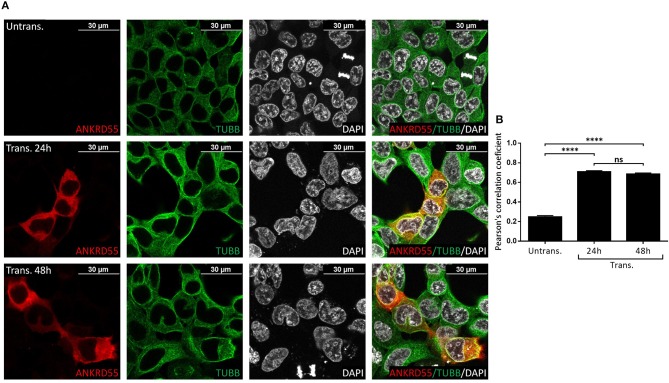
Recombinant ANKRD55 isoform 001 colocalizes with endogenous β-tubulin in HEK293 cells. **(A)** Representative microphotographs of immunostaining for ANKRD55 (anti-ANKRD55 Ab; red), tubulin beta chain (TUBB Ab; green) and nuclei (DAPI; white) in untransfected (Untrans.) and transfected (Trans. 24 h or 48 h) HEK293 cells with ANKRD55 isoform 001. **(B)** Colocalization quantification using Pearson's correlation coefficient. Data are mean ± SEM (*n* = 10 cellular ROIs/condition), *p* ≤ 0.0001 (^****^), and not significant (ns) comparing three conditions, Mann-Whitney test.

**Figure 9:**
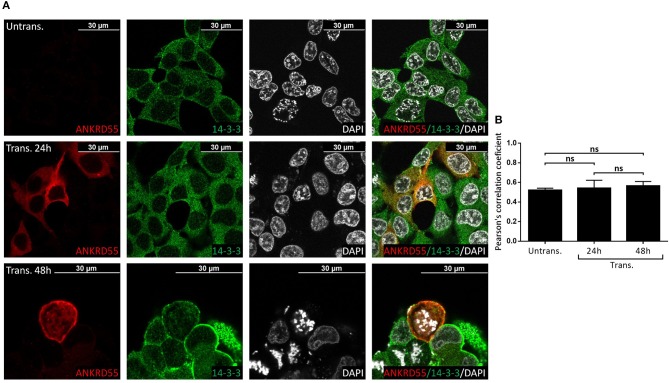
Recombinant ANKRD55 isoform 001 colocalizes with endogenous 14-3-3 proteins in HEK293 cells. **(A)** Representative microphotographs of immunostaining for ANKRD55 (anti-ANKRD55 Ab; red), 14-3-3 (14-3-3 Ab; green) and nuclei (DAPI; white) in untransfected (Untrans.) and transfected (Trans. 24 h or 48 h) HEK293 cells with ANKRD55 isoform 001. **(B)** Colocalization quantification using Pearson's correlation coefficient. Data are mean ± SEM (*n* = 10 cellular ROIs/condition), not significant (ns) comparing three conditions, Mann-Whitney test.

**Figure 10:**
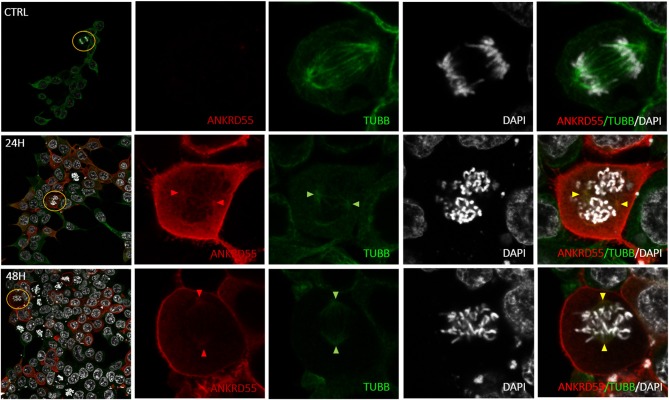
Colocalization of nuclear recombinant ANKRD55 iosoform 001 with endogenous β-tubulin in HEK293 cells following antigen retrieval treatment. Representative microphotographs of immunostaining for ANKRD55 (FLAG Ab; red), tubulin beta chain (TUBB Ab; green) and nuclei (DAPI; white) in non-transfected (CTRL) and transfected (24 h or 48 h) HEK293 cells with ANKRD55 isoform 001 subjected to antigen retrieval treatment. Original image included zoom-in region (yellow), the signal intensity in zoom-in images was increased. Colocalization of ANKRD55 with TUBB in the mitotic spindle is indicated with colored triangles.

Proteins that interact tend to reside within the same or adjacent subcellular compartments (46). Fluorescence colocalization microscopy can thus be used to assess potential links between distinct molecules (47). Subcellular localization of endogenous and recombinant ANKRD55 using anti-ANKRD55 Ab with a selection of interacting partners was analyzed to verify sharing of specific subcellular localizations. Under conditions used for visualization, anti-ANKRD55 antibody detected ANKRD55 in transfected cells but not in untransfected cells (Supplementary Figure 4). However, endogenous ANKRD55 could be visualized in untransfected cells using over-/underexposure Look-up Table scale (Supplementary Figure 5). In the IF Figures, we include the Pearson's correlation coefficient for colocalization of RPS3, TUBB, 14-3-3 and VIM with both endogenous ANKRD55 (Untrans.) and recombinant ANKRD55 (Trans.) at 24 and 48 h after transfection (B panels in Figures 7–9, Supplementary Figure 6). The results showed a highly significant colocalization (*p* ≤ 0.001) between recombinant ANKRD55 isoform 001 and either RPS3 (Figure 7) or β-tubulin isoforms (Figure 8) in both the cytoplasm and cell membrane. Correlation of colocalization between 14-3-3 and ANKRD55 was not higher with recombinant ANKRD55 (Figure 9) than with the endogenous one, and that with VIM only increased at 48 h (Supplementary Figure 6), suggesting already-high occupancy of these proteins with the endogenous ANKRD55. Recombinant ANKRD55 colocalization with β-tubulin and 14-3-3 isoforms remained constant, but decreased over time after transfection with RPS3 (Figures 7–9) and increased with VIM (Supplementary Figure 6). As shown in Figures 7, 8 ANKRD55 was poorly detectable in the nucleus, despite its presence in biochemically or sucrose-gradient obtained nuclear fractions (Figure 2). The procedure was modified to include an antigen retrieval treatment step to try to increase the fluorescent signal of the nuclei. This modification was successful when used in combination with a highly sensitive anti-FLAG Ab and was applied to study the nuclear colocalization of overexpressed ANKRD55 together with β-tubulin isoforms (Figure 10) and VIM (Supplementary Figure 7). After antigen retrieval treatment, recombinant ANKRD55 became visible in the nuclei of cells in division, though these cells exhibited a lower fluorescence intensity signal than not-dividing cells. Quantification was not performed due to the small sample size. ANKRD55 positively colocalized with diffuse β-tubulin isoforms in the nucleus and specifically with the mitotic spindle (Figure 10). It also colocalized with diffuse VIM in the nucleus without an associated specific structure (Supplementary Figure 7).

### ANKRD55 Is a Phosphoprotein

An important subset of proteins identified in this interactome analysis belong to the 14-3-3 protein family. 14-3-3 proteins are ubiquitously expressed phosphoserine/phosphothreonine (pS/pT)-binding proteins that are members of a large family of isoforms (48). In general, they bind to their ligands through RSXpS/TXP or RXXXpS/TXP sequences (49), but they can also bind their ligands in a phosphorylation-independent manner (50, 51). Thus, we determined whether ANKRD55 was phosphorylated. Our primary sources for this step were phosphoproteome repositories, such as PhosphoSitePlus (35) and dbPAF (36) databases. Moreover, 14-3-3-Pred web server (37) was used to predict putative 14-3-3 binding phosphosites on ANKRD55. Two out of ninety predicted phosphorylation sites (S436 and S597) fulfill the cut-off (Supplementary Table 9). We identified specific serine and threonine phosphorylation sites in overexpressed and immunoprecipitated ANKRD55 isoform 001 from HEK293 nuclear and total proteins extracts by TiO_2_-based phosphopeptide enrichment prior to nLC–MS/MS analysis. The phosphorylation sites at T6 and S436 in ANKRD55 were confirmed and the S475 site was found in nuclear and total protein extracts previously identified in mouse ANKRD55 (Table 2). In conclusion, the data revealed five phosphorylation sites in human ANKRD55 at T6, T11, T189, S436 and S475. 14-3-3-Pred web server predicted with the highest score S436 as 14-3-3 binding phosphosite.

**Table 2:** Summary of identified Ser/Thr Phosphorylation Sites in ANKRD55.

**Position**	**Peptides**	**Species**	**References**	**Sources**	**Identified phosphorylation sites**
T6	MMRQAtMDFSTPS	Human	(52)	PhosphoSitePlus	N/T
T11	QATMDFStPSVFDQQ	Human	(52)	PhosphoSitePlus	
T189	GADPtLVDK	Human	(53)	dbPAF	
S436	TQsLPPITLGNNFLTASHR	Human	(54)	dbPAF	T
S473	MAQRSQKsRSEQDLL	Mouse	(55, 56)	PhosphoSitePlus	
S475	QRSQKSRsEQDLLNN	Mouse	(55–60)	PhosphoSitePlus	N/T

*The first five columns show the phosphorylation sites identified in the phosphoproteome repositories including position and sequence of the peptide, species in which it was found, reference for the experiment data and database. The last column represents our own experimental data identifying specific ANKRD55 phosphosites: N, nuclear extracts and T, total protein extracts*.

## Discussion

*ANKRD55* emerged as the nearest annotated gene of which expression is affected by a series of *cis*-eQTL risk SNPs that were identified through multiple GWAS and other studies in diverse multifactorial—predominantly autoimmune, diseases (1–17). However, the biological function(s) of ANKRD55 has (have) not been elucidated. Ankyrin repeats function as versatile scaffolds for protein–protein interactions. ANKRD55 isoform 001, the main focus of this study, contains nine ankyrin repeats and consequently should take part in diverse PPIs as has been reported for other members of the ANK family (21–27). Similar to naturally produced ANKRD55 (18), overexpressed recombinant ANKRD55 is present in the nuclei and cytosol. We therefore characterized the ANKRD55 protein interactome from both TPE and sucrose-gradient-purified NE. The limitations of using co-immunoprecipitation in overexpressing cell lines to determine protein interactions should be noted, as this approach may lead to non-specific interactions and prevents spatial or temporal analysis of protein interactions (61). A larger group of ANKRD55-interacting partners was present in total protein extracts than in nuclear extracts with 148 vs. 22 proteins consistently identified over three independent replicates. Functional analysis suggested that ANKRD55-protein partners from nuclear (eight and seven proteins) and total protein extracts (fifty-two and forty-three proteins) are related to nucleotide and ATP binding, respectively. 30% of proteins from the nuclear interactome are related to RNA binding.

Experiments were performed to validate top-scoring partners of ANKRD55 by western blot, reverse IP and/or colocalization in IF microscopy. Four distinct 14-3-3 proteins (epsilon, beta/alpha, eta and gamma) were identified in total cell extracts. 14-3-3 colocalized with ANKRD55 in IF microscopy, and ANKRD55 was detected in reverse IP of 14-3-3ε, the major ANKRD55 partner (Table 1). Being pS/pT-binding proteins, their presence led us to identify and confirm five phosphorylation sites in ANKRD55 with S436 as most likely 14-3-3 binding phosphosite. 14-3-3 proteins modulate the action of proteins that are involved in cell cycle and transcriptional control, signal transduction, intracellular trafficking and regulation of ion channels (62) through a variety of mechanisms: (i) direct conformational change of the target protein; (ii) physical occlusion of sequence-specific or structural features; and (iii) scaffolding that anchors proteins within close proximity of one another (63).

Eleven ribosomal proteins (RPs) were identified in ANKRD55 interactomes. Of these, RPS3 was identified in both nuclear and total protein extract interactomes as one of the top 10 hits, and was confirmed by western blot of IPs and by colocalization with ANKRD55 by IF microscopy. ANKRD55 was also detected in reverse IP of RPS3. Although RPs are known for playing an essential role in ribosome assembly and protein translation, their ribosome-independent functions are also increasingly being appreciated (64). RPS3, specifically, has been shown to induce apoptosis by collaborating with E2F1 (65). Phospho-RPS3 translocates into the nucleus and upregulates prosurvival gene expression via association with NF-κB in non-small cell lung cancer cells (66, 67). RPS3 is also involved in immune signaling by selectively modulating NF-κB target gene expression. Given the fact that RPS3 also activates the p53 tumor suppressive pathway (68), this RP is regarded as one of the most fascinating RP with pivotal multifunctions (69).

ANKRD55 colocalized with TUBB in IF microscopy, and their interaction was confirmed by western blot; ANKRD55 was also identified in reverse IPs of TUBB. Inspection by IF microscopy revealed that ANKRD55 is detectable in the nucleus mainly during mitosis. Throughout the different phases of mitosis, β-tubulin presented a specific marking in the mitotic spindle that colocalized with ANKRD55. Other proteins were reproducibly identified in the nuclear ANKRD55 interactome that have been reported to fulfill roles associated with mitotic spindle dynamics or structure. These include SMC3 and SMC1A that are members of the cohesin complex that is crucial for chromatid cohesion. By holding the two sister chromatids together from their formation during replication until their separation in anaphase, cohesin creates a counterforce to the pulling of the mitotic spindle, which allows correct chromosome alignment and segregation (70). The association between the ANKRD55-interacting proteins and the mitotic spindle is also supported by the presence of RPS3 protein which was reported to be involved in spindle dynamics (71). Similarly, Ran GTPase binding, enriched in the GO analysis, has been related to the spindle formation (72). MCM3, 6 and 7, DNA helicases involved in the initiation of DNA replication, were also found in ANRD55 interactomes. Of these, MCM7 is known to co-localize with tubulin in mitotic cells with MCM7 depletion resulting in aberrant mitosis. This is in line with observations that MCM7 exerts certain functions on spindle formation to prevent cytokinesis during early mitosis by regulating CDK1 activity (73).

GO categories from total protein extracts were enriched in nuclear transport terms providing a possible explanation for the nucleocytoplasmic transport of ANKRD55, which seems to be mediated by the importins/exportins transport system enriched in the interactome (CSE1L, XPO1, IPO4, XPOT, IPO5, NUP160, NUP205, KPNA2, COPG1, CDK5, YWHAH, CLTC). However, a number of AR-containing proteins, such as IκBα, ASPP2, and GABPβ, have been reported to enter the nucleus via an unknown mechanism independent of a canonical nuclear localization signal (74). As demonstrated by Lu et al. (75), this mechanism is characterized by the presence of components of the RanGDP/AR pathway which represents a general importin-independent nuclear import pathway frequently used by AR-containing proteins (75). Ran GTPase binding, which obtained high significance in the GO, is known to regulate protein trafficking through the nuclear envelope and is an active component of both nuclear transport systems.

In conclusion, the large number of protein interactors identified in this study suggests that ANKRD55 may act as a scaffold, probably exerting function(s) in the formation or architecture of multiple protein complexes, that may be regulated via (de)phosphorylation reactions occurring at up to 5 sites in the protein.

## Data Availability

The mass spectrometry proteomics data have been deposited to the ProteomeXchange Consortium via the PRIDE partner repository with the dataset identifier PXD013332.

## Author Contributions

NU, SB, JM, IA, MA, FE, and KV designed and performed the experiments. NU and KV wrote the manuscript. IA, MA, JM, and FE critically reviewed the manuscript.

### Conflict of Interest Statement

The authors declare that the research was conducted in the absence of any commercial or financial relationships that could be construed as a potential conflict of interest.

## Footnotes

^1^http://interactome.baderlab.org/
