# Supplementary material for: Interactome of the Autoimmune Risk Protein ANKRD55
Source: Front Immunol. 2019 Sep 18;10:2067. doi: 10.3389/fimmu.2019.02067 (PMC6759997; doi:10.3389/fimmu.2019.02067)
Supplement: Supplementary file 1 [file Data_Sheet_1.docx]

*Supplementary Material*

Interactome of the autoimmune risk protein ANKRD55

*Nerea Ugidos^1,2,^*, Jorge Mena^1,2,^*, Sara Baquero^1,2^, Iraide Alloza^1,2^, Mikel Azkargorta^3^, Felix Elortza^3^ and Koen Vandenbroeck^1,2,4^*

***^1^*** *Neurogenomiks Group, Department of Neuroscience, University of the Basque Country (UPV/EHU), Leioa, Spain*

*^2^ Achucarro Basque Center for Neuroscience, Leioa, Spain*

*^3^ Proteomics Platform, CIC bioGUNE, CIBERehd, ProteoRed-ISCIII, Bizkaia Science and Technology Park, 48160 Derio, Spain*

*^4^ IKERBASQUE, Basque Foundation for Science, Bilbao, Spain*

*Shared first authorship

**Correspondence:** Koen Vandenbroeck [k.vandenbroeck@ikerbasque.org](mailto:k.vandenbroeck@ikerbasque.org)


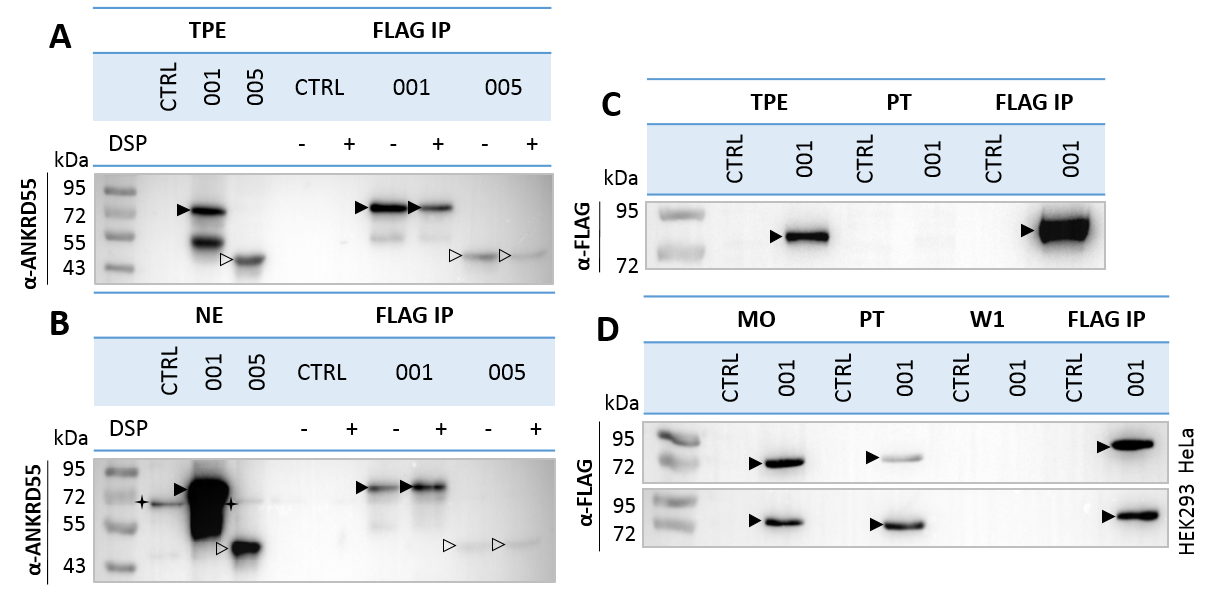


Supplementary Figure 1. Overexpression and immunoprecipitation of recombinant ANKRD55 isoforms. (A, B, D) HEK293 and (C, D) HeLa cells were transfected with FLAG-tagged isoforms (001) and (005) of ANKRD55. Cells were treated +/- DSP, when indicated, and IP was performed on enriched nuclear fractions (NE), total protein extracts (TPE) or membranous organelles fraction (MO) after 48h of transfection. Recombinant ANKRD55 isoforms were detected in NE, TPE, MO and eluted fractions (FLAG IP) with specific anti-ANKRD55 Ab (Sigma-Aldrich) or anti-FLAG Ab by WB. As negative control cells were cultivated under the same conditions, but without transfection agent (CTRL). Specific bands corresponding to overexpressed isoforms 001 and 005 are indicated by black and white arrowheads, respectively. Black cross marks endogenous ANKRD55 isoform 001. PT, pass through fraction; W1, first washing step.


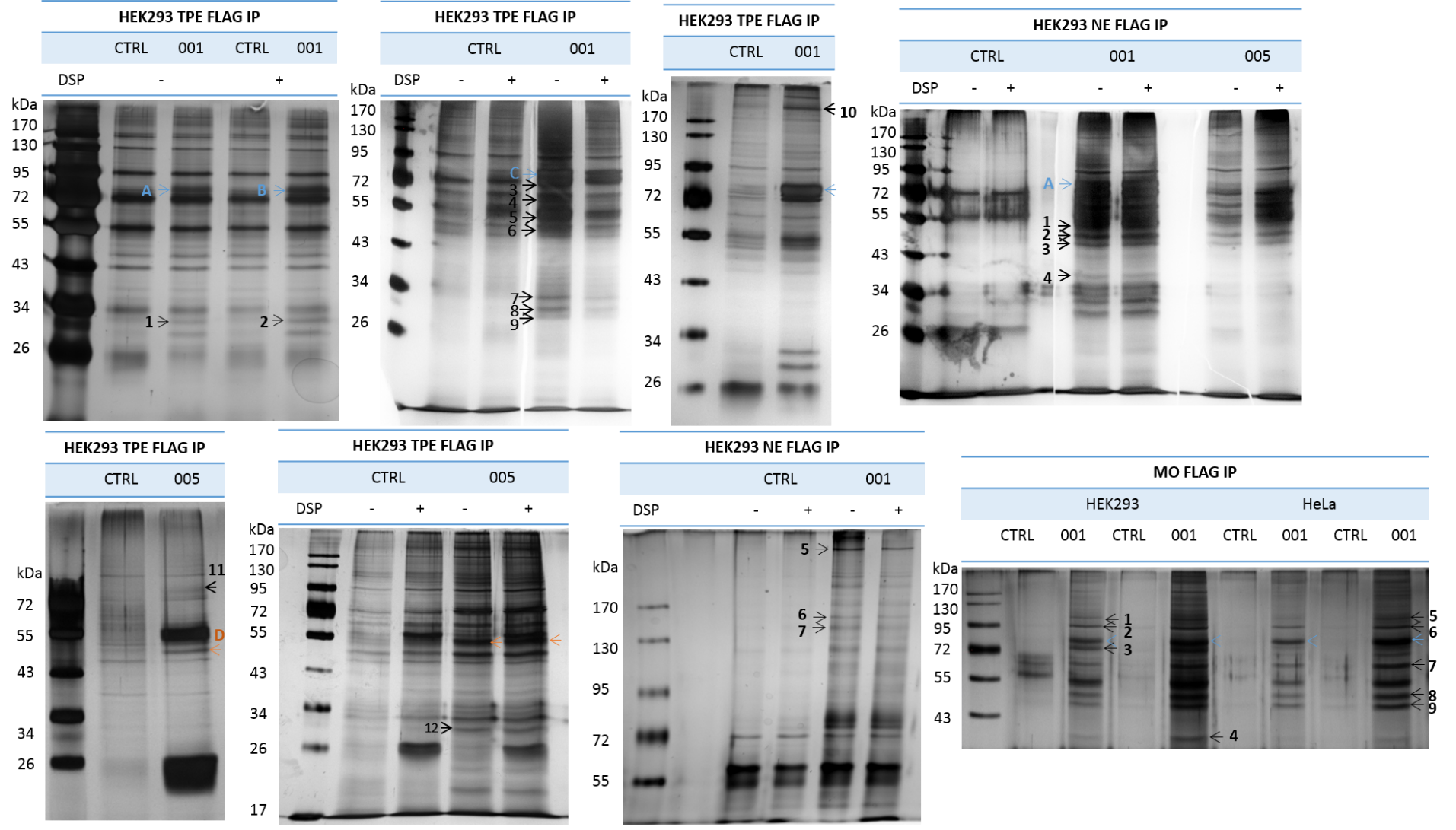


Supplementary Figure 2. Silver-staining analysis of ANKRD55 complexes. Silver-stained SDS-PAGE showing ANKRD55 IP with FLAG resin from membranous organelles fraction (MO), total protein extracts (TPE), and nuclear fractions (NE) of HEK293 and HeLa cells expressing FLAG-tagged ANKRD55 isoforms (001) and (005). As negative control cells were cultivated under the same conditions, but without transfection agent (CTRL). The proteins identified using nLC-MS/MS are listed in Table 6 and marked by black arrowheads. The recombinant ANKRD55 001 and 005 are indicated by blue and red arrowheads, respectively.


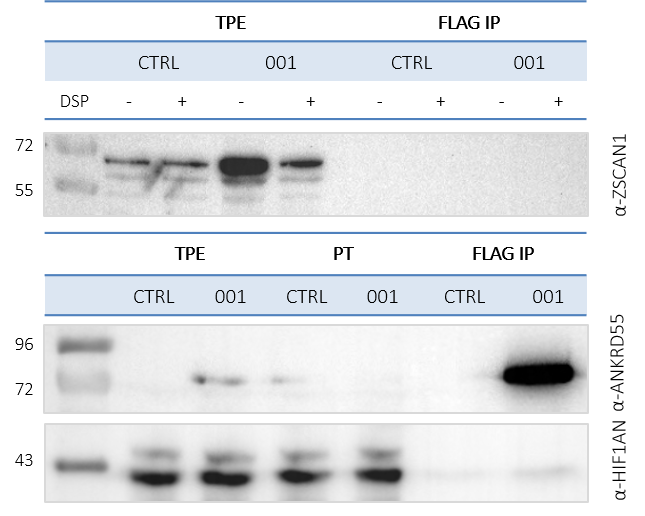


Supplementary Figure 3. Validation of ANKRD55-interacting partners from BioGrid database. HEK293 cells were transfected with FLAG-tagged isoform 001 of ANKRD55 and IPed from total protein extracts (TPE) after 48h of transfection. Endogenous HIF1AN and overexpressed ANKRD55 were detected in TPE, pass-through (PT), and elution with specific Ab by WB.


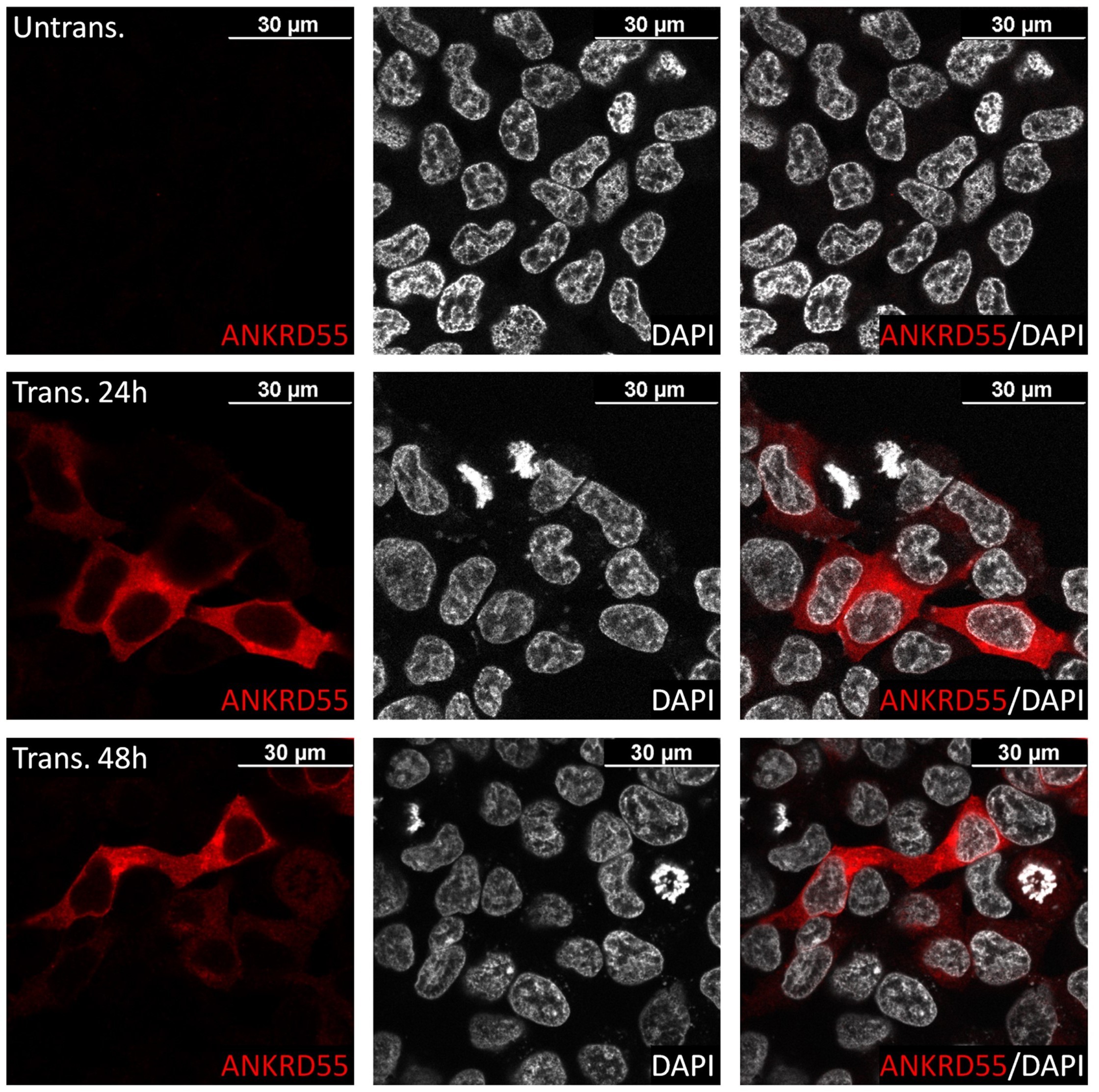


**Supplementary Figure 4. ANKRD55 isoform 001 expression in HEK293 cells.**

Representative microphotographs of immunostaining for ANKRD55 (anti-ANKRD55 Ab; red) and nuclei (DAPI; white) in untransfected (Untrans.) and transfected (Trans. 24h or 48h) HEK293 cells with ANKRD55 isoform 001.

**
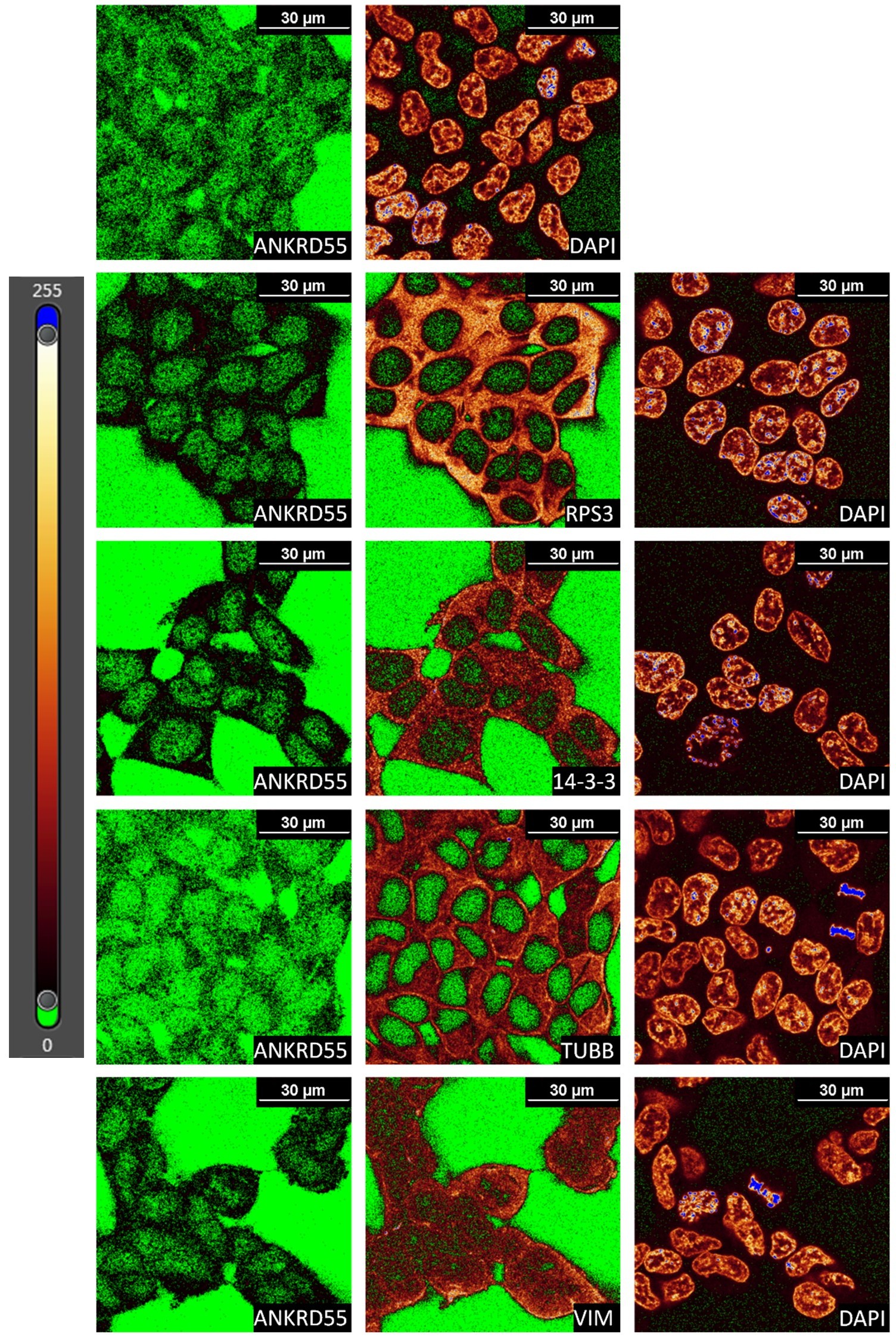
**

**Supplementary Figure 5. Endogenous ANKRD55, RPS3, 14-3-3, TUBB and VIM in HEK293 cells.**

Microphotographs **from Figures 7, 8, 9** and **Supplementary Figure 4 & 6** of immunostaining for ANKRD55, RPS3, 14-3-3, TUBB, VIM and DAPI in untransfected HEK293 cells showed as over-/underexposure LUT (*“Look-Up Table”*) scale (Leica Application Suite Advanced Fluorescence) software. This mode assigns a green color to underexposed areas in the image with a grey value of ≤0 and a blue color to overexposed areas with grey values >255 (for 8 bit).

**
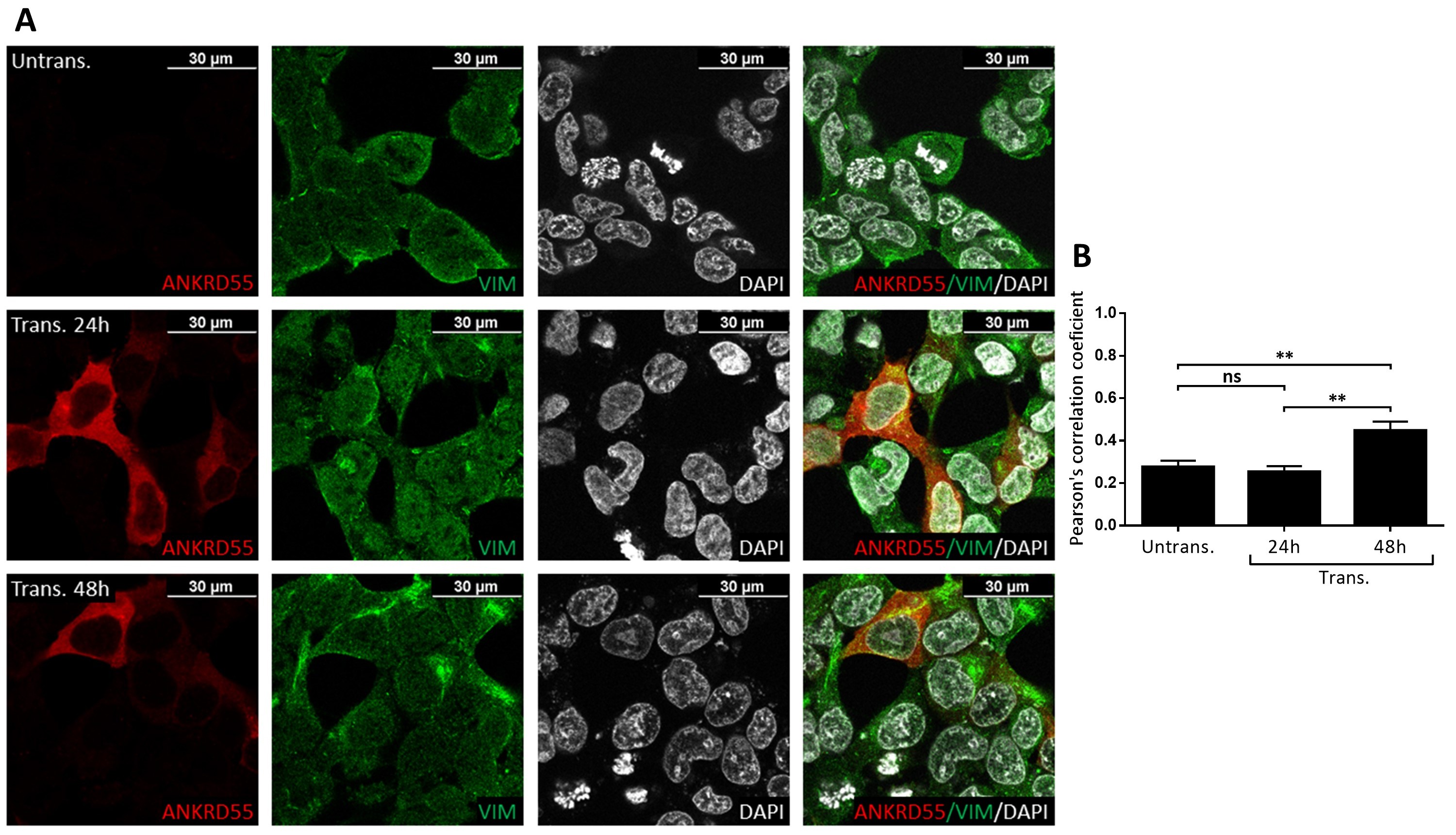
**

Supplementary Figure 6. Recombinant ANKRD55 isoform 001 colocalizes with endogenous VIM in HEK293 cells. (A) Representative microphotographs of immunostaining for ANKRD55 (anti-ANKRD55 Ab; red), vimentin (VIM Ab; green) and nuclei (DAPI; white) in untransfected (Untrans.) and transfected (Trans. 24h or 48h) HEK293 cells with ANKRD55 isoform 001. **(B)** Colocalization quantification using Pearson’s correlation coefficient. Data are mean ± SEM (n=10 cellular ROIs/condition), p≤0.01 (**), and not significant (ns) comparing three conditions, Mann-Whitney test.


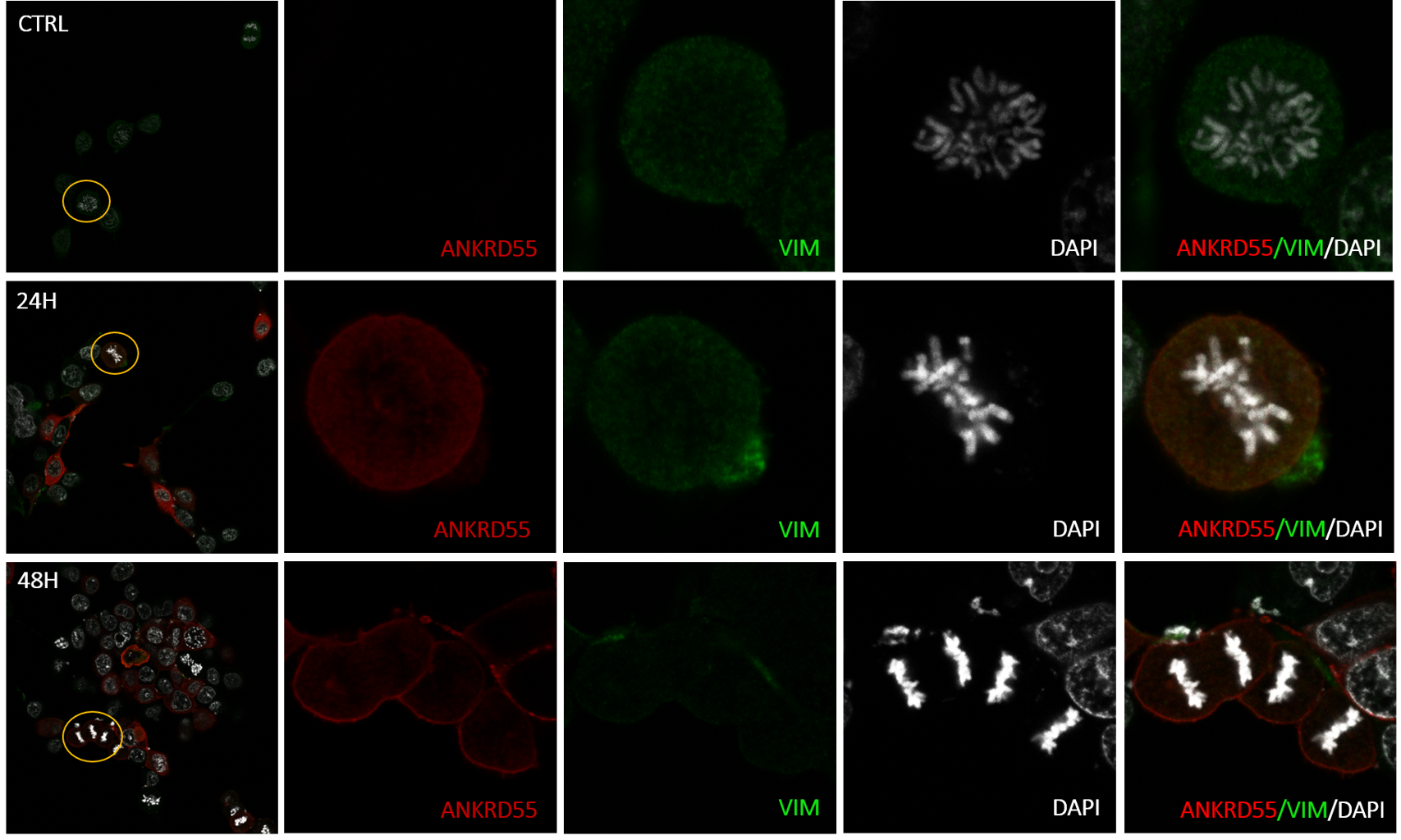


Supplementary Figure 7. Recombinant ANKRD55 colocalizes with endogenous nuclear VIM in HEK293 cells following antigen retrieval treatment. Representative microphotographs of immunostaining for ANKRD55 (FLAG Ab; red), vimentin (VIM Ab; green) and nuclei (DAPI; white) in non-transfected (CTRL) and transfected 24h (24h) and 48h (48h) HEK293 cells with ANKRD55 isoform 001 subjected to antigen retrieval treatment. Original image included zoon-in region (yellow), the signal intensity in zoom-in images was increased.

**Supplementary Table 1. Identified ANKRD55-interacting proteins from three different cell extracts by silver staining and nLC-MS/MS analysis**

|  | IP | Band | Gene symbol | Accession | MW (kDa) | Protein name | Peptides |
| --- | --- | --- | --- | --- | --- | --- | --- |
| TOTAL PROTEIN EXTRACTS | HEK293 001 | A | ANKRD55 | Q3KP44 | 68.41 | Ankyrin repeat domain-containing protein 55 | 12 |
|  |  |  | HSPA9 | P38646 | 73.68 | Stress-70 protein, mitochondrial | 4 |
|  |  | B | ANKRD55 | Q3KP44 | 68.41 | Ankyrin repeat domain-containing protein 55 | 9 |
|  |  |  | HSPA9 | P38646 | 73.68 | Stress-70 protein, mitochondrial | 4 |
|  |  | 1 | YWHAE | P62258 | 29.17 | 14-3-3 protein epsilon | 4 |
|  |  | 2 | YWHAE | P62258 | 29.17 | 14-3-3 protein epsilon | 3 |
|  |  | C | ANKRD55 | Q3KP44 | 68.41 | Ankyrin repeat domain-containing protein 55 | 19 |
|  |  |  | HSPA5 | P11021 | 72.28 | 78 kDa glucose-regulated protein | 8 |
|  |  | 3 | LMNB1 | P20700 | 66.408 | Lamin-B1 | 21 |
|  |  |  | HSPA1A | P0DMV8 | 70 | Heat shock 70 kDa protein 1A/1B | 16 |
|  |  |  | LMNB2 | Q03252 | 69.948 | Lamin B2 | 15 |
|  |  |  | RPN1 | P04843 | 68.569 | Dolichyl-diphosphooligosaccharide--protein glycosyltransferase subunit 1 | 4 |
|  |  | 4 | VIM | P08670 | 53.65 | Vimentin | 13 |
|  |  |  | CCT4 | P50991 | 57.924 | T-complex protein 1 subunit delta | 7 |
|  |  |  | PKM | P14618 | 57.937 | Pyruvate kinase PKM | 3 |
|  |  |  | CCT7 | Q99832 | 59.367 | T-complex protein 1 subunit eta | 2 |
|  |  |  | TCP1 | P17987 | 60.344 | T-complex protein 1 subunit alpha | 2 |
|  |  | 5 | TUBB | P07437 | 49.6 | Tubulin beta chain | 10 |
|  |  |  | TUBA1B | P68363 | 50.1 | Tubulin alpha-1B chain | 9 |
|  |  |  | ATP5B | P06576 | 56.56 | ATP synthase subunit beta, mitochondrial | 7 |
|  |  | 6 | EEF1A1 | P68104 | 50.14 | Elongation factor 1-alpha 1 | 4 |
|  |  |  | TUFM | P49411 | 49.542 | Elongation factor Tu, mitochondrial | 4 |
|  |  |  | EIF4A1 | P60842 | 46.15 | Eukaryotic initiation factor 4A-I | 2 |
|  |  | 7 | YWHAE | P62258 | 29.174 | 14-3-3 protein epsilon | 13 |
|  |  |  | GNB2L1 | P63244 | 35.07 | Guanine nucleotide-binding protein subunit beta-2-like 1 Receptor of activated protein C kinase 1 | 4 |
|  |  |  | RPS3 | P23396 | 26.688 | 40S ribosomal protein S3 | 4 |
|  |  | 8 | YWHAZ | P63104 | 27.745 | 14-3-3 protein zeta/delta | 25 |
|  |  |  | PHB | P35232 | 29.8 | Prohibitin | 3 |
|  |  |  | SLC25A5 | P05141 | 32.852 | ADP/ATP translocase 2 | 6 |
|  |  |  | YWHAE | P62258 | 29.174 | 14-3-3 protein epsilon | 3 |
|  |  |  | SLC25A6 | P12236 | 32.866 | ADP/ATP translocase 3 | 5 |
|  |  |  | YWHAH | Q04917 | 28.219 | 14-3-3 protein eta | 3 |
|  |  |  | YWHAB | P31946 | 28.082 | 14-3-3 protein beta/alpha | 3 |
|  |  |  | YWHAQ | P27348 | 27.76 | 14-3-3 protein theta | 2 |
|  |  |  | YWHAG | P61981 | 28.3 | 14-3-3 protein gamma | 3 |
|  |  | 9 | YWHAZ | P63104 | 27.745 | 14-3-3 protein zeta/delta | 6 |
|  |  |  | YWHAG | P61981 | 28.3 | 14-3-3 protein gamma | 5 |
|  |  |  | PHB | P35232 | 29.8 | Prohibitin | 3 |
|  |  |  | YWHAH | Q04917 | 28.219 | 14-3-3 protein eta | 6 |
|  |  |  | YWHAQ | P27348 | 27.76 | 14-3-3 protein theta | 5 |
|  |  |  | SLC25A5 | P05141 | 32.852 | ADP/ATP translocase 2 | 6 |
|  |  |  | SLC25A6 | P12236 | 32.866 | ADP/ATP translocase 3 | 6 |
|  |  |  | YWHAB | P31946 | 28.082 | 14-3-3 protein beta/alpha | 6 |
|  |  | 10 | CAD | P27708 | 242.984 | CAD protein | 18 |
|  |  |  | MYH10 | P35580 | 228.99 | Myosin-10 | 4 |
|  |  |  | USP9X | Q93008 | 292.28 | Probable ubiquitin carboxyl-terminal hydrolase FAF-X | 3 |
|  |  |  | GCN1L1 | Q92616 | 292.75 | Translational Activator GCN1 | 3 |
|  |  |  | PRKDC | P78527 | 469.089 | DNA-dependent protein kinase catalytic subunit | 3 |
|  |  |  | FASN | P49327 | 273.427 | Fatty acid synthase | 2 |
|  | HEK293 005 | 11 | HSPA5 | P11021 | 72.28 | 78 kDa glucose-regulated protein | 2 |
|  |  | D | ANKRD55 | Q3KP44 | 36.9 | Isoform 2 Ankyrin repeat domain-containing protein 55 | 13 |
|  |  |  | EEF1A1 | P68104 | 50.1 | Elongation factor 1-alpha 1 | 2 |
|  |  | 12 | YWHAZ | P63104 | 27.745 | 14-3-3 protein zeta/delta | 5 |
| NUCLEAR EXTRACTS | HEK293 001 | A | ANKRD55 | Q3KP44 | 68.41 | Ankyrin repeat domain-containing protein 55 | 13 |
|  |  |  | HSPA5 | P11021 | 72.28 | 78 kDa glucose-regulated protein | 2 |
|  |  | 1 | TUBB | P07437 | 49.60 | Tubulin beta chain | 12 |
|  |  |  | TUBB4B | P68371 | 49.80 | Tubulin beta-4B chain | 12 |
|  |  |  | TUBB4A | P04350 | 49.60 | Tubulin beta-4A chain | 10 |
|  |  |  | TUBA1B | P68363 | 50.10 | Tubulin alpha-1B chain | 9 |
|  |  |  | ATP5B | P06576 | 56.56 | ATP synthase subunit beta, mitochondrial | 5 |
|  |  | 2 | EIF4A1 | P60842 | 46.15 | Eukaryotic initiation factor 4A-I | 5 |
|  |  |  | EEF1A1 | P68104 | 50.14 | Elongation factor 1-alpha 1 | 4 |
|  |  |  | DDOST | P39656 | 50.801 | Dolichyl-diphosphooligosaccharide--protein glycosyltransferase 48 kDa subunit | 5 |
|  |  |  | EIF4A3 | P38919 | 46.871 | Eukaryotic initiation factor 4A-III | 5 |
|  |  |  | RPL3 | P39023 | 46.109 | 60S ribosomal protein L3 | 3 |
|  |  |  | TUBB | P07437 | 49.60 | Tubulin beta chain | 3 |
|  |  |  | EEF1G | P26641 | 50.119 | Elongation factor 1-gamma | 3 |
|  |  | 3 | ACTB | P60709 | 41.737 | Actin, cytoplasmic 1 | 10 |
|  |  |  | CKB | P12277 | 42.644 | Creatine kinase B-type | 5 |
|  |  |  | OAT | P04181 | 48.53 | Ornithine aminotransferase, mitochondrial | 3 |
|  |  |  | CS | O75390 | 51.712 | Citrate synthase, mitochondrial | 3 |
|  |  |  | PAICS | P22234 | 47.079 | Multifunctional protein ADE2 | 3 |
|  |  | 4 | GAPDH | P04406 | 36.05 | Glyceraldehyde-3-phosphate dehydrogenase | 3 |
|  |  |  | RPLP0 | P05388 | 36.05 | 60S acidic ribosomal protein P0 | 3 |
|  |  | 5 | PRKDC | P78527 | 469.089 | DNA-dependent protein kinase catalytic subunit | 23 |
|  |  | 6 | SMC1A | Q14683 | 143.23 | Structural maintenance of chromosomes protein 1A | 6 |
|  |  | 7 | SMC3 | Q9UQE7 | 141.54 | Structural maintenance of chromosomes protein 3 | 13 |
|  |  |  | DHX9 | Q08211 | 140.95 | ATP-dependent RNA helicase A | 6 |
| MEMBRANOUS ORGANELLES  nousNOUS ORGANELLES | HEK293 001 | 1 | HSP90B1 | P14625 | 92.469 | Endoplasmin | 6 |
|  |  |  | GANAB | Q14697 | 106.874 | Neutral alpha-glucosidase AB | 4 |
|  |  | 2 | HSP90AB1 | P08238 | 83.264 | Heat shock protein HSP 90-beta | 26 |
|  |  |  | HSP90AA1 | P07900 | 84.66 | Heat shock protein HSP 90-alpha | 23 |
|  |  |  | MCM5 | P33992 | 82.286 | DNA replication licensing factor MCM5 | 3 |
|  |  | 3 | HSPA8 | P11142 | 70.89 | Heat shock cognate 71 kDa protein | 21 |
|  |  |  | HSPA1A | P0DMV8 | 70.052 | Heat shock 70 kDa protein 1A | 17 |
|  |  |  | HSPA9 | P38646 | 73.68 | Stress-70 protein, mitochondrial | 8 |
|  |  |  | SLC25A13 | Q9UJS0 | 74.176 | Calcium-binding mitochondrial carrier protein Aralar2 | 5 |
|  |  |  | HSPA5 | P11021 | 72.28 | 78 kDa glucose-regulated protein | 3 |
|  |  |  | XRCC6 | P12956 | 69.843 | X-ray repair cross-complementing protein 6 | 3 |
|  |  |  | IFT74 | Q96LB3 | 69.239 | Intraflagellar transport protein 74 homolog | 3 |
|  |  | 4 | GAPDH | P04406 | 36.05 | Glyceraldehyde-3-phosphate dehydrogenase | 13 |
|  | HeLa 001 | 5 | HSP90B1 | P14625 | 92.469 | Endoplasmin | 9 |
|  |  |  | EEF2 | P13639 | 95.338 | Elongation factor 2 | 4 |
|  |  |  | GANAB | Q14697 | 106.874 | Neutral alpha-glucosidase AB | 4 |
|  |  | 6 | HSP90AB1 | P08238 | 83.264 | Heat shock protein HSP 90-beta | 16 |
|  |  |  | HSP90AA1 | P07900 | 84.66 | Heat shock protein HSP 90-alpha | 10 |
|  |  | 7 | PKM | P14618 | 57.937 | Pyruvate kinase PKM | 14 |
|  |  |  | HSPD1 | P10809 | 61.055 | 60 kDa heat shock protein, mitochondrial | 7 |
|  |  |  | TCP1 | P17987 | 60.344 | T-complex protein 1 subunit alpha | 4 |
|  |  | 8 | EIF4A1 | P60842 | 46.154 | Eukaryotic initiation factor 4A-I | 5 |
|  |  |  | EEF1A1 | P68104 | 50.14 | Elongation factor 1-alpha 1 | 3 |
|  |  | 9 | TUFM | P49411 | 49.542 | Elongation factor Tu, mitochondrial | 10 |
|  |  |  | EIF4A1 | P60842 | 46.154 | Eukaryotic initiation factor 4A-I | 6 |

Supplementary Table 2. ANKRD55-interacting proteins identified in total protein extracts of HEK293 cells using nLC-MS/MS

|  | Gene symbol | Accession | MW (kDa) | Protein | NSAF | |
| --- | --- | --- | --- | --- | --- | --- |
|  |  |  |  |  | **ANKRD55** | **CTRL** |
|  | CFL1 | P23528 | 18.502 | Cofilin-1 | 0.31 | 0.00 |
|  | CCT8 | P50990 | 59.621 | T-complex protein 1 subunit theta | 0.31 | 0.05 |
|  | ATP5O | P48047 | 23.277 | ATP synthase subunit O, mitochondrial | 0.31 | 0.00 |
|  | PPP2R1A | P30153 | 65.309 | Serine/threonine-protein phosphatase 2A 65 kDa regulatory subunit A alpha isoform | 0.31 | 0.03 |
|  | RPS9 | P46781 | 22.591 | 40S ribosomal protein S9 | 0.31 | 0.00 |
|  | DNAJA1 | P31689 | 44.868 | DnaJ homolog subfamily A member 1 | 0.30 | 0.00 |
|  | SLC25A12 | O75746 | 74.762 | Calcium-binding mitochondrial carrier protein Aralar1 | 0.30 | 0.00 |
|  | HSPA9 | P38646 | 73.68 | Stress-70 protein, mitochondrial | 0.29 | 0.07 |
|  | SRPRB | Q9Y5M8 | 29.702 | Signal recognition particle receptor subunit beta | 0.28 | 0.00 |
|  | CAD | P27708 | 242.984 | CAD protein | 0.27 | 0.00 |
|  | RPS3A | P61247 | 29.945 | 40S ribosomal protein S3a | 0.27 | 0.00 |
|  | GNB2L1 | P63244 | 35.077 | Guanine nucleotide-binding protein subunit beta-2-like 1 | 0.27 | 0.00 |
|  | SFXN1 | Q9H9B4 | 35.619 | Sideroflexin-1 | 0.27 | 0.06 |
|  | DPM1 | O60762 | 29.634 | Dolichol-phosphate mannosyltransferase subunit 1 | 0.27 | 0.00 |
|  | ARL1 | P40616 | 20.418 | ADP-ribosylation factor-like protein 1 | 0.27 | 0.00 |
|  | SLC25A1 | P53007 | 34.013 | Tricarboxylate transport protein, mitochondrial | 0.27 | 0.00 |
|  | RPL10 | P27635 | 24.604 | 60S ribosomal protein L10 | 0.27 | 0.00 |
|  | PRKDC | P78527 | 469.089 | DNA-dependent protein kinase catalytic subunit | 0.26 | 0.01 |
|  | RPLP0 | P05388 | 36.05 | 60S acidic ribosomal protein P0 | 0.25 | 0.00 |
|  | RPN1 | P04843 | 68.569 | Dolichyl-diphosphooligosaccharide--protein glycosyltransferase subunit 1 | 0.24 | 0.04 |
|  | PPP2R2A | P63151 | 51.692 | Serine/threonine-protein phosphatase 2A 55 kDa regulatory subunit B alpha isoform | 0.24 | 0.00 |
|  | RPS2 | P15880 | 31.324 | 40S ribosomal protein S2 | 0.24 | 0.00 |
|  | SLC25A3 | Q00325 | 40.095 | Phosphate carrier protein, mitochondrial | 0.24 | 0.05 |
|  | VDAC2 | P45880 | 31.567 | Voltage-dependent anion-selective channel protein 2 | 0.24 | 0.00 |
|  | TTC26 | A0AVF1 | 64.178 | Intraflagellar transport protein 56 | 0.24 | 0.00 |
|  | EIF4A3 | P38919 | 46.871 | Eukaryotic initiation factor 4A-III | 0.23 | 0.00 |
|  | DDX3X | O00571 | 73.243 | ATP-dependent RNA helicase DDX3X | 0.23 | 0.00 |
|  | C1QBP | Q07021 | 31.362 | Complement component 1 Q subcomponent-binding protein, mitochondrial | 0.22 | 0.00 |
|  | RCN2 | Q14257 | 36.876 | Reticulocalbin-2 | 0.22 | 0.00 |
|  | RBM39 | Q14498 | 59.38 | RNA-binding protein 39 | 0.22 | 0.00 |
|  | CDK5 | Q00535 | 33.304 | Cyclin-dependent-like kinase 5 | 0.22 | 0.00 |
|  | OAT | P04181 | 48.53 | Ornithine aminotransferase, mitochondrial | 0.21 | 0.00 |
|  | AIFM1 | O95831 | 66.901 | Apoptosis-inducing factor 1, mitochondrial | 0.21 | 0.00 |
|  | RAB5C | P51148 | 23.483 | Ras-related protein Rab-5C | 0.20 | 0.00 |
|  | RPL7A | P62424 | 29.996 | 60S ribosomal protein L7a | 0.20 | 0.00 |
|  | ATP1A1 | P05023 | 112.896 | Sodium/potassium-transporting ATPase subunit alpha-1 | 0.20 | 0.00 |
|  | SERPINH1 | P50454 | 46.44 | Serpin H1 | 0.20 | 0.00 |
|  | CSE1L | P55060 | 110.417 | Exportin-2 | 0.20 | 0.00 |
|  | IRS4 | O14654 | 133.768 | Insulin receptor substrate 4 | 0.19 | 0.00 |
|  | RARS | P54136 | 75.379 | Arginine--tRNA ligase, cytoplasmic | 0.19 | 0.00 |
|  | IFT74 | Q96LB3 | 69.239 | Intraflagellar transport protein 74 homolog | 0.19 | 0.00 |
|  | LRPPRC | P42704 | 157.905 | Leucine-rich PPR motif-containing protein, mitochondrial | 0.19 | 0.03 |
|  | NCL | P19338 | 76.614 | Nucleolin | 0.18 | 0.06 |
|  | RTCB | Q9Y3I0 |  | tRNA-splicing ligase RtcB homolog | 0.18 | 0.04 |
|  | SSR1 | P43307 | 32.235 | Translocon-associated protein subunit alpha | 0.18 | 0.00 |
|  | CLTC | Q00610 | 191.615 | Clathrin heavy chain 1 | 0.18 | 0.02 |
|  | PDHB | P11177 | 39.233 | Pyruvate dehydrogenase E1 component subunit beta, mitochondrial | 0.17 | 0.00 |
|  | ATAD3B | Q5T9A4 | 72.573 | ATPase family AAA domain-containing protein 3B | 0.17 | 0.00 |
|  | ETFA | P13804 | 35.08 | Electron transfer flavoprotein subunit alpha, mitochondrial | 0.17 | 0.00 |
|  | PYCRL | Q53H96 | 28.663 | Pyrroline-5-carboxylate reductase 3 | 0.17 | 0.00 |
|  | PTBP1 | P26599 | 57.221 | Polypyrimidine tract-binding protein 1 | 0.17 | 0.00 |
|  | XPO1 | O14980 | 123.386 | Exportin-1 | 0.17 | 0.00 |
|  | HSD17B12 | Q53GQ0 | 34.324 | Very-long-chain 3-oxoacyl-CoA reductase | 0.16 | 0.00 |
|  | FARSA | Q9Y285 | 57.564 | Phenylalanine--tRNA ligase alpha subunit | 0.16 | 0.04 |
|  | CBSL | P0DN79 | 60.587 | Cystathionine beta-synthase-like protein | 0.16 | 0.00 |
|  | ALDH18A1 | P54886 | 87.302 | Delta-1-pyrroline-5-carboxylate synthase | 0.15 | 0.00 |
|  | IARS | P41252 | 144.498 | Isoleucine--tRNA ligase, cytoplasmic | 0.15 | 0.00 |
|  | MCM6 | Q14566 | 92.889 | DNA replication licensing factor MCM6 | 0.15 | 0.00 |
|  | XPOT | O43592 | 109.964 | Exportin-T | 0.15 | 0.00 |
|  | ATP5C1 | P36542 | 32.996 | ATP synthase subunit gamma, mitochondrial | 0.14 | 0.00 |
|  | SMC1A | Q14683 | 143.23 | Structural maintenance of chromosomes protein 1A | 0.14 | 0.00 |
|  | DNAJA2 | O60884 | 45.746 | DnaJ homolog subfamily A member 2 | 0.14 | 0.00 |
|  | MCM3 | P25205 | 90.981 | DNA replication licensing factor MCM3 | 0.14 | 0.00 |
|  | AARS | P49588 | 106.81 | Alanine--tRNA ligase, cytoplasmic | 0.14 | 0.00 |
|  | BSG | P35613 | 42.2 | Basigin | 0.14 | 0.00 |
|  | DNM2 | P50570 | 98.064 | Dynamin-2 | 0.14 | 0.00 |
|  | CTPS1 | P17812 | 66.69 | CTP synthase 1 | 0.13 | 0.00 |
|  | CAND1 | Q86VP6 | 136.376 | Cullin-associated NEDD8-dissociated protein 1 | 0.13 | 0.00 |
|  | DDX21 | Q9NR30 | 87.344 | Nucleolar RNA helicase 2 | 0.13 | 0.00 |
|  | ATP2A2 | P16615 | 114.757 | Sarcoplasmic/endoplasmic reticulum calcium ATPase 2 | 0.13 | 0.00 |
|  | SLC1A5 | Q15758 | 56.598 | Neutral amino acid transporter B(0) | 0.12 | 0.00 |
|  | TIMM44 | O43615 | 51.35 | Mitochondrial import inner membrane translocase subunit TIM44 | 0.12 | 0.00 |
|  | RPL4 | P36578 | 47.697 | 60S ribosomal protein L4 | 0.12 | 0.00 |
|  | FARSB | Q9NSD9 | 66.116 | Phenylalanine--tRNA ligase beta subunit | 0.12 | 0.00 |
|  | FASN | P49327 | 273.427 | Fatty acid synthase | 0.12 | 0.01 |
|  | PSMC3 | P17980 | 49.204 | Proteasome 26S Subunit, ATPase 3 | 0.12 | 0.00 |
|  | IMPDH2 | P12268 | 55.805 | Inosine-5'-monophosphate dehydrogenase 2 | 0.12 | 0.00 |
|  | EFTUD2 | Q15029 | 109.436 | 116 kDa U5 small nuclear ribonucleoprotein component | 0.11 | 0.00 |
|  | MMS19 | Q96T76 | 113.29 | MMS19 nucleotide excision repair protein homolog | 0.11 | 0.00 |
|  | IPO4 | Q8TEX9 | 118.715 | Importin-4 | 0.11 | 0.00 |
|  | MARS | P56192 | 101.116 | Methionine--tRNA ligase, cytoplasmic | 0.11 | 0.00 |
|  | KPNA2 | P52292 | 57.862 | Importin subunit alpha-1 | 0.11 | 0.00 |
|  | ATXN10 | Q9UBB4 | 53.489 | Ataxin-10 | 0.11 | 0.00 |
|  | GCN1L1 | Q92616 | 292.75 | Translational activator GCN1 | 0.10 | 0.00 |
|  | SNRNP200 | O75643 | 244.508 | U5 small nuclear ribonucleoprotein 200 kDa helicase | 0.10 | 0.01 |
|  | NOP56 | O00567 | 66.05 | Nucleolar protein 56 | 0.10 | 0.00 |
|  | ABCD3 | P28288 | 75.476 | ATP-binding cassette sub-family D member 3 | 0.10 | 0.00 |
|  | ARAF | P10398 | 67.585 | Serine/threonine-protein kinase A-Raf | 0.09 | 0.00 |
|  | COPG1 | Q9Y678 | 97.718 | Coatomer subunit gamma-1 | 0.09 | 0.00 |
|  | TRIP13 | Q15645 | 48.551 | Pachytene checkpoint protein 2 homolog | 0.09 | 0.00 |
|  | SLC27A4 | Q6P1M0 | 72.064 | Long-chain fatty acid transport protein 4 | 0.09 | 0.00 |
|  | RARS2 | Q5T160 | 65.505 | Probable arginine--tRNA ligase, mitochondrial | 0.08 | 0.00 |
|  | LARS | Q9P2J5 | 134.466 | Leucine--tRNA ligase, cytoplasmic | 0.08 | 0.00 |
|  | MIB1 | Q86YT6 | 110.136 | E3 ubiquitin-protein ligase MIB1 | 0.08 | 0.00 |
|  | ADSL | P30566 | 54.889 | Adenylosuccinate lyase | 0.08 | 0.00 |
|  | PRMT5 | O14744 | 72.684 | Protein arginine N-methyltransferase 5 | 0.07 | 0.00 |
|  | MYBBP1A | Q9BQG0 | 148.855 | Myb-binding protein 1A | 0.07 | 0.00 |
|  | USP9X | Q93008 | 292.28 | Probable ubiquitin carboxyl-terminal hydrolase FAF-X | 0.07 | 0.00 |
|  | ADCK3 | Q8NI60 | 71.95 | Atypical kinase ADCK3, mitochondrial | 0.07 | 0.00 |
|  | UPF1 | Q92900 | 124.345 | Regulator of nonsense transcripts 1 | 0.06 | 0.00 |
|  | DYNC1H1 | Q14204 | 532.408 | Cytoplasmic dynein 1 heavy chain 1 | 0.06 | 0.00 |
|  | WDR6 | Q9NNW5 | 121.725 | WD repeat-containing protein 6 | 0.06 | 0.00 |
|  | USP7 | Q93009 | 128.302 | Ubiquitin carboxyl-terminal hydrolase 7 | 0.06 | 0.00 |
|  | CNOT1 | A5YKK6 | 266.939 | CCR4-NOT transcription complex subunit 1 | 0.06 | 0.00 |
|  | FANCI | Q9NVI1 | 149.324 | Fanconi anemia group I protein | 0.06 | 0.00 |
|  | ACLY | P53396 | 120.839 | ATP-citrate synthase | 0.06 | 0.00 |
|  | MTHFD1L | Q6UB35 | 105.79 | Monofunctional C1-tetrahydrofolate synthase, mitochondrial | 0.05 | 0.00 |
|  | PRPF8 | Q6P2Q9 | 273.6 | Pre-mRNA-processing-splicing factor 8 | 0.05 | 0.00 |
|  | IPO5 | O00410 | 123.63 | Importin-5 | 0.05 | 0.00 |
|  | VARS | P26640 | 140.476 | Valine--tRNA ligase | 0.05 | 0.00 |
|  | SF3B3 | Q15393 | 135.577 | Splicing factor 3B subunit 3 | 0.05 | 0.00 |
|  | GANAB | Q14697 | 106.874 | Neutral alpha-glucosidase AB | 0.05 | 0.00 |
|  | PNPLA6 | Q8IY17 | 149.995 | Neuropathy target esterase | 0.05 | 0.00 |
|  | UBE3C | Q15386 | 123.923 | Ubiquitin-protein ligase E3C | 0.04 | 0.00 |
|  | DDB1 | Q16531 | 126.968 | DNA damage-binding protein 1 | 0.04 | 0.00 |
|  | EIF3A | Q14152 | 166.569 | Eukaryotic translation initiation factor 3 subunit A | 0.04 | 0.00 |
|  | NUP160 | Q12769 | 162.121 | Nuclear pore complex protein Nup160 | 0.04 | 0.00 |
|  | TLN1 | Q9Y490 | 269.767 | Talin-1 | 0.04 | 0.00 |
|  | UBR5 | O95071 | 309.352 | E3 ubiquitin-protein ligase UBR5 | 0.04 | 0.00 |
|  | PI4KA | P42356 | 236.83 | Phosphatidylinositol 4-kinase alpha | 0.03 | 0.00 |
|  | NCAPD3 | P42695 | 168.891 | Condensin-2 complex subunit D3 | 0.03 | 0.00 |
|  | FLNA | P21333 | 280.739 | Filamin-A | 0.03 | 0.00 |
|  | MDN1 | Q9NU22 | 632.82 | Midasin | 0.03 | 0.00 |
|  | NUP205 | Q92621 | 227.922 | Nuclear pore complex protein Nup205 | 0.03 | 0.00 |
|  | LRBA | P50851 | 319.108 | Lipopolysaccharide-responsive and beige-like anchor protein | 0.02 | 0.00 |
|  | ACACA | Q13085 | 265.554 | Acetyl-CoA carboxylase 1 | 0.02 | 0.00 |

Supplementary Table 3. Identification of ANKRD55-interacting partners in a minimum of two replicates of nuclear and total protein extracts by nLC-MS/MS

|  | Gene symbol | Accession | MW (kDa) | Protein | NSAF | |
| --- | --- | --- | --- | --- | --- | --- |
|  |  |  |  |  | **ANKRD55** | **CTRL** |
| TOTAL PROTEIN EXTRACTS | TUBB | P07437 | 49.60 | Tubulin beta chain | 2.23 | 1.48 |
|  | TUBB4B | P68371 | 49.80 | Tubulin beta-4B chain | 1.92 | 1.35 |
|  | TUBA1B | P68363 | 50.152 | Tubulin alpha-1B chain | 1.60 | 0.00 |
|  | YWHAZ | P63104 | 27.745 | 14-3-3 protein zeta/delta | 1.60 | 0.67 |
|  | YWHAQ | P27348 | 27.76 | 14-3-3 protein theta | 1.32 | 0.67 |
|  | HSPA1A | P0DMV8 | 63.89 | Isoform 2 of Heat shock 70 kDa protein 1A/1B | 1.31 | 0.77 |
|  | TUBB2A | Q13885 | 49.907 | Tubulin beta-2A chain | 1.14 | 0.00 |
|  | EIF4A1 | P60842 | 46.15 | Eukaryotic initiation factor 4A-I | 1.10 | 0.81 |
|  | HSPD1 | P10809 | 61.055 | 60 kDa heat shock protein, mitochondrial | 0.75 | 0.19 |
|  | RPS18 | P62269 | 17.719 | 40S ribosomal protein S18 | 0.66 | 1.44 |
|  | SLC25A6 | P12236 | 32.866 | ADP/ATP translocase 3 | 0.61 | 0.00 |
|  | RUVBL1 | Q9Y265 | 50.22 | RuvB-like 1 | 0.55 | 0.24 |
|  | CCT2 | P78371 | 57.488 | T-complex protein 1 subunit beta | 0.50 | 0.10 |
|  | ARF3 | P61204 | 20.601 | ADP-ribosylation factor 3 | 0.47 | 0.00 |
|  | PKM | P14618 | 57.937 | Pyruvate kinase PKM | 0.47 | 0.21 |
|  | TUFM | P49411 | 49.542 | Elongation factor Tu, mitochondrial | 0.47 | 0.24 |
|  | PPIA | P62937 | 18.012 | Peptidyl-prolyl cis-trans isomerase A | 0.46 | 0.33 |
|  | CCT6A | P40227 | 58.024 | T-complex protein 1 subunit zeta | 0.37 | 0.00 |
|  | PRDX1 | Q06830 | 22.11 | Peroxiredoxin-1 | 0.37 | 1.10 |
|  | RPS16 | P62249 | 16.445 | 40S ribosomal protein S16 | 0.37 | 0.38 |
|  | UQCRC2 | P22695 | 48.443 | Cytochrome b-c1 complex subunit 2, mitochondrial | 0.36 | 0.00 |
|  | RPS14 | P62263 | 16.273 | 40S ribosomal protein S14 | 0.35 | 0.36 |
|  | RPS4X | P62701 | 29.598 | 40S ribosomal protein S4, X isoform | 0.31 | 0.21 |
|  | ATAD3A | Q9NVI7 | 71.369 | ATPase family AAA domain-containing protein 3A | 0.29 | 0.17 |
|  | CCT5 | P48643 | 59.671 | T-complex protein 1 subunit epsilon | 0.29 | 0.20 |
|  | DDX39A | O00148 | 49.13 | ATP-dependent RNA helicase DDX39A | 0.28 | 0.00 |
|  | ALDH1B1 | P30837 | 57.206 | Aldehyde dehydrogenase X, mitochondrial | 0.27 | 0.00 |
|  | DARS | P14868 | 57.136 | Aspartate--tRNA ligase, cytoplasmic | 0.26 | 0.00 |
|  | HSP90AB1 | P08238 | 83.264 | Heat shock protein HSP 90-beta | 0.26 | 0.53 |
|  | CCT7 | Q99832 | 59.367 | T-complex protein 1 subunit eta | 0.26 | 0.10 |
|  | DHX9 | Q08211 | 140.95 | ATP-dependent RNA helicase A | 0.26 | 0.09 |
|  | ILF2 | Q12905 | 43.06 | Interleukin enhancer-binding factor 2 | 0.24 | 0.00 |
|  | RPS20 | P60866 | 13.373 | 40S ribosomal protein S20 | 0.24 | 0.00 |
|  | RPS11 | P62280 | 18.431 | 40S ribosomal protein S11 | 0.22 | 0.00 |
|  | EEF2 | P13639 | 95.338 | Elongation factor 2 | 0.22 | 0.06 |
|  | RPN2 | P04844 | 69.284 | Dolichyl-diphosphooligosaccharide--protein glycosyltransferase subunit 2 | 0.21 | 0.00 |
|  | DDX17 | Q92841 | 80.272 | Probable ATP-dependent RNA helicase DDX17 | 0.21 | 0.00 |
|  | CCT3 | P49368 | 60.534 | T-complex protein 1 subunit gamma | 0.20 | 0.00 |
|  | NDUFA13 | Q9P0J0 | 16.698 | NADH dehydrogenase [ubiquinone] 1 alpha subcomplex subunit 13 | 0.19 | 0.00 |
|  | PRPS1 | P60891 | 34.834 | Ribose-phosphate pyrophosphokinase 1 | 0.19 | 0.00 |
|  | SLC25A10 | Q9UBX3 | 31.282 | Mitochondrial dicarboxylate carrier | 0.18 | 0.00 |
|  | MTHFD1 | P11586 | 101.559 | C-1-tetrahydrofolate synthase, cytoplasmic | 0.18 | 0.23 |
|  | DDOST | P39656 | 50.801 | Dolichyl-diphosphooligosaccharide--protein glycosyltransferase 48 kDa subunit | 0.17 | 0.00 |
|  | RPL11 | P62913 | 20.12 | 60S ribosomal protein L11 | 0.17 | 0.00 |
|  | HNRNPH1 | P31943 | 49.229 | Heterogeneous nuclear ribonucleoprotein H | 0.16 | 0.00 |
|  | CDIPT | O14735 | 23.539 | CDP-diacylglycerol--inositol 3-phosphatidyltransferase | 0.15 | 0.00 |
|  | PABPC1 | P11940 | 70.67 | Polyadenylate-binding protein 1 | 0.15 | 0.00 |
|  | OTUB1 | Q96FW1 | 31.284 | Ubiquitin thioesterase OTUB1 | 0.14 | 0.00 |
|  | MCM5 | P33992 | 82.286 | DNA replication licensing factor MCM5 | 0.14 | 0.00 |
|  | SRSF3 | P84103 | 19.33 | Serine/arginine-rich splicing factor 3 | 0.13 | 0.00 |
|  | EPRS | P07814 | 170.591 | Bifunctional glutamate/proline--tRNA ligase | 0.13 | 0.14 |
|  | TARDBP | Q13148 | 44.74 | TAR DNA-binding protein 43 | 0.13 | 0.00 |
|  | ANXA2P2 | A6NMY6 | 38.659 | Putative annexin A2-like protein | 0.13 | 0.00 |
|  | TIMM23 | O14925 | 21.943 | Mitochondrial import inner membrane translocase subunit Tim23 | 0.13 | 0.00 |
|  | FAF2 | Q96CS3 | 52.623 | FAS-associated factor 2 | 0.13 | 0.00 |
|  | PSMD2 | Q13200 |  | 26S proteasome non-ATPase regulatory subunit 2 | 0.12 | 0.06 |
|  | DIMT1 | Q9UNQ2 | 35.236 | Probable dimethyladenosine transferase | 0.12 | 0.00 |
|  | PCMT1 | P22061 | 24.636 | Protein-L-isoaspartate(D-aspartate) O-methyltransferase | 0.12 | 0.00 |
|  | RQCD1 | Q92600 | 33.631 | Cell differentiation protein RCD1 homolog | 0.12 | 0.00 |
|  | KPNB1 | Q14974 | 97.17 | Importin subunit beta-1 | 0.12 | 0.06 |
|  | YBX1 | P67809 | 35.924 | Nuclease-sensitive element-binding protein 1 | 0.12 | 0.00 |
|  | SURF4 | O15260 | 30.394 | Surfeit locus protein 4 | 0.12 | 0.00 |
|  | PSMC4 | P43686 | 47.366 | 26S protease regulatory subunit 6B | 0.12 | 0.13 |
|  | TUBG1 | P23258 | 51.17 | Tubulin gamma-1 chain | 0.12 | 0.00 |
|  | SRM | P19623 | 102.335 | Spermidine synthase | 0.11 | 0.00 |
|  | STUB1 | Q9UNE7 | 34.856 | E3 ubiquitin-protein ligase CHIP | 0.11 | 0.00 |
|  | ACOT8 | O14734 | 35.914 | Acyl-coenzyme A thioesterase 8 | 0.11 | 0.00 |
|  | PSMC6 | P62333 | 44.173 | 26S protease regulatory subunit 10B | 0.10 | 0.00 |
|  | HNRNPU | Q00839 | 90.584 | Heterogeneous nuclear ribonucleoprotein U | 0.10 | 0.00 |
|  | PSMD3 | O43242 | 60.978 | 26S proteasome non-ATPase regulatory subunit 3 | 0.10 | 0.00 |
|  | HAX1 | O00165 | 31.621 | HCLS1-associated protein X-1 | 0.09 | 0.00 |
|  | PSMD12 | O00232 | 52.904 | 26S proteasome non-ATPase regulatory subunit 12 | 0.09 | 0.00 |
|  | SEC61A1 | P61619 | 52.265 | Protein transport protein Sec61 subunit alpha isoform 1 | 0.09 | 0.00 |
|  | VDAC3 | Q9Y277 | 30.659 | Voltage-dependent anion-selective channel protein 3 | 0.09 | 0.00 |
|  | MSH2 | P43246 | 104.743 | DNA mismatch repair protein Msh2 | 0.09 | 0.00 |
|  | SLC25A18 | Q9H1K4 | 33.849 | Mitochondrial glutamate carrier 2 | 0.09 | 0.00 |
|  | HNRNPA0 | Q13151 | 30.841 | Heterogeneous nuclear ribonucleoprotein A0 | 0.09 | 0.00 |
|  | SMC3 | Q9UQE7 | 141.54 | Structural maintenance of chromosomes protein 3 | 0.09 | 0.00 |
|  | COPB1 | P53618 | 107.142 | Coatomer subunit beta | 0.09 | 0.00 |
|  | DHRS7B | Q6IAN0 | 35.119 | Dehydrogenase/reductase SDR family member 7B | 0.09 | 0.00 |
|  | RPL8 | P62917 | 28.025 | 60S ribosomal protein L8 | 0.08 | 0.00 |
|  | AASDHPPT | Q9NRN7 | 35.776 | L-aminoadipate-semialdehyde dehydrogenase-phosphopantetheinyl transferase | 0.08 | 0.00 |
|  | SCO2 | O43819 | 29.81 | Protein SCO2 homolog, mitochondrial | 0.08 | 0.00 |
|  | TRIM28 | Q13263 | 88.55 | Transcription intermediary factor 1-beta | 0.08 | 0.07 |
|  | GALK1 | P51570 | 42.272 | Galactokinase | 0.08 | 0.00 |
|  | MATR3 | P43243 | 94.623 | Matrin-3 | 0.08 | 0.00 |
|  | IFT52 | Q9Y366 | 49.706 | Intraflagellar transport protein 52 homolog | 0.08 | 0.00 |
|  | TIMMDC1 | Q9NPL8 | 32.178 | Complex I assembly factor TIMMDC1, mitochondrial | 0.08 | 0.00 |
|  | RFC3 | P40938 | 40.556 | Replication factor C subunit 3 | 0.07 | 0.00 |
|  | EIF3L | Q9Y262 | 66.727 | Eukaryotic translation initiation factor 3 subunit L | 0.07 | 0.00 |
|  | CDC45 | O75419 | 65.569 | Cell division control protein 45 homolog | 0.07 | 0.00 |
|  | WDR36 | Q8NI36 | 105.322 | WD repeat-containing protein 36 | 0.07 | 0.00 |
|  | LBR | Q14739 | 70.703 | Lamin-B receptor | 0.07 | 0.00 |
|  | COPA | P53621 | 138.346 | Coatomer subunit alpha | 0.07 | 0.00 |
|  | STT3A | P46977 | 80.53 | Dolichyl-diphosphooligosaccharide--protein glycosyltransferase subunit STT3A | 0.07 | 0.00 |
|  | AGK | Q53H12 | 47.137 | Acylglycerol kinase, mitochondrial | 0.07 | 0.00 |
|  | DHX15 | O43143 | 90.933 | Pre-mRNA-splicing factor ATP-dependent RNA helicase DHX15 | 0.07 | 0.00 |
|  | KHSRP | Q92945 | 73.115 | Far upstream element-binding protein 2 | 0.06 | 0.00 |
|  | EI24 | O14681 | 38.965 | Etoposide-induced protein 2.4 homolog | 0.06 | 0.00 |
|  | UMPS | P11172 | 52.222 | Uridine 5'-monophosphate synthase | 0.06 | 0.00 |
|  | GARS | P41250 | 83.166 | Glycine--tRNA ligase | 0.06 | 0.00 |
|  | POLD1 | P28340 | 123.631 | DNA polymerase delta catalytic subunit | 0.06 | 0.00 |
|  | FAR1 | Q8WVX9 | 59.357 | Fatty acyl-CoA reductase 1 | 0.06 | 0.00 |
|  | MCM4 | P33991 | 96.558 | DNA replication licensing factor MCM4 | 0.06 | 0.00 |
|  | DDX1 | Q92499 | 82.432 | ATP-dependent RNA helicase DDX1 | 0.06 | 0.00 |
|  | UNC45A | Q9H3U1 | 103.077 | Protein unc-45 homolog A | 0.06 | 0.00 |
|  | COPB2 | P35606 | 102.487 | Coatomer subunit beta' | 0.06 | 0.00 |
|  | HSPH1 | Q92598 | 96.865 | Heat shock protein 105 kDa | 0.05 | 0.00 |
|  | SARS | P49591 | 58.777 | Serine--tRNA ligase, cytoplasmic | 0.05 | 0.00 |
|  | TELO2 | Q9Y4R8 | 91.747 | Telomere length regulation protein TEL2 homolog | 0.05 | 0.00 |
|  | TRAP1 | Q12931 | 80.11 | Heat shock protein 75 kDa, mitochondrial | 0.05 | 0.00 |
|  | NSUN2 | Q08J23 | 86.471 | tRNA (cytosine(34)-C(5))-methyltransferase | 0.05 | 0.00 |
|  | PTPN1 | P18031 | 49.967 | Tyrosine-protein phosphatase non-receptor type 1 | 0.05 | 0.00 |
|  | NCLN | Q969V3 | 62.974 | Nicalin | 0.05 | 0.00 |
|  | ASNS | P08243 | 64.37 | Asparagine synthetase [glutamine-hydrolyzing] | 0.05 | 0.00 |
|  | MSH6 | P52701 | 152.786 | DNA mismatch repair protein Msh6 | 0.04 | 0.00 |
|  | HSP90B1 | P14625 | 92.469 | Endoplasmin | 0.04 | 0.00 |
|  | CCDC8 | Q9H0W5 | 59.374 | Coiled-coil domain-containing protein 8 | 0.04 | 0.00 |
|  | TNPO1 | Q92973 | 102.355 | Transportin-1 | 0.04 | 0.00 |
|  | POLR2B | P30876 | 133.897 | DNA-directed RNA polymerase II subunit RPB2 | 0.04 | 0.00 |
|  | IQGAP1 | P46940 | 189.252 | Ras GTPase-activating-like protein IQGAP1 | 0.04 | 0.00 |
|  | DIS3 | Q9Y2L1 | 109.003 | Exosome complex exonuclease RRP44 | 0.04 | 0.00 |
|  | AARS2 | Q5JTZ9 | 107.34 | Alanine--tRNA ligase, mitochondrial | 0.04 | 0.00 |
|  | IPO9 | Q96P70 | 115.963 | Importin-9 | 0.03 | 0.00 |
|  | TFRC | P02786 | 84.871 | Transferrin receptor protein 1 | 0.03 | 0.00 |
|  | IARS2 | Q9NSE4 | 113.792 | Isoleucine--tRNA ligase, mitochondrial | 0.03 | 0.00 |
|  | DHX37 | Q8IY37 | 129.545 | Probable ATP-dependent RNA helicase DHX37 | 0.03 | 0.00 |
|  | RPTOR | Q8N122 | 149.038 | Regulatory-associated protein of mTOR | 0.03 | 0.00 |
|  | ESYT2 | A0FGR8 | 102.357 | Extended synaptotagmin-2 | 0.03 | 0.00 |
|  | SMC4 | Q9NTJ3 | 147.182 | Structural maintenance of chromosomes protein 4 | 0.03 | 0.00 |
|  | IPO7 | O95373 | 119.517 | Importin-7 | 0.03 | 0.00 |
|  | PPP6R3 | Q5H9R7 | 97.669 | Serine/threonine-protein phosphatase 6 regulatory subunit 3 | 0.02 | 0.00 |
|  | WDR11 | Q9BZH6 | 136.685 | WD repeat-containing protein 11 | 0.02 | 0.00 |
|  | USO1 | O60763 | 107.895 | General vesicular transport factor p115 | 0.02 | 0.00 |
|  | MYO1B | O43795 | 131.985 | Unconventional myosin-Ib | 0.02 | 0.00 |
|  | RFC1 | P35251 | 128.255 | Replication factor C subunit 1 | 0.02 | 0.00 |
|  | DHX30 | Q7L2E3 | 133.938 | Putative ATP-dependent RNA helicase DHX30 | 0.02 | 0.00 |
|  | TARBP1 | Q13395 | 181.675 | Probable methyltransferase TARBP1 | 0.02 | 0.00 |
|  | HEATR1 | Q9H583 | 242.37 | HEAT repeat-containing protein 1 | 0.02 | 0.00 |
|  | TBC1D4 | O60343 | 146.563 | TBC1 domain family member 4 | 0.02 | 0.00 |
|  | BTAF1 | O14981 | 206.887 | TATA-binding protein-associated factor 172 | 0.02 | 0.00 |
|  | HUWE1 | Q7Z6Z7 | 481.891 | E3 ubiquitin-protein ligase HUWE1 | 0.01 | 0.00 |
|  | ANAPC1 | Q9H1A4 | 216.5 | Anaphase-promoting complex subunit 1 | 0.01 | 0.00 |
|  | NUP188 | Q5SRE5 | 196.043 | Nucleoporin NUP188 homolog | 0.01 | 0.00 |
| NUCLEAR EXTRACTS | HSPA1A | P0DMV8 | 63.89 | Isoform 2 of Heat shock 70 kDa protein 1A/1B | 1.59 | 0.29 |
|  | EIF4A1 | P60842 | 46.15 | Eukaryotic initiation factor 4A-I | 0.89 | 0.16 |
|  | DHX9 | Q08211 | 140.95 | ATP-dependent RNA helicase A | 0.82 | 0.24 |
|  | NONO | Q15233 | 54.232 | Non-POU domain-containing octamer-binding protein | 0.73 | 0.26 |
|  | HSPA8 | P11142 | 70.89 | Heat shock cognate 71 kDa protein | 0.70 | 0.14 |
|  | TUBB | P07437 | 49.60 | Tubulin beta chain | 0.62 | 0.00 |
|  | RPL22 | P35268 | 14.787 | 60S ribosomal protein L22 | 0.60 | 0.40 |
|  | RUVBL2 | Q9Y230 | 51.157 | RuvB-like 2 | 0.59 | 0.05 |
|  | DDX47 | Q9H0S4 | 50.647 | Probable ATP-dependent RNA helicase DDX47 | 0.36 | 0.00 |
|  | MATR3 | P43243 | 94.623 | Matrin-3 | 0.36 | 0.17 |
|  | HNRNPH3 | P31942 | 36.926 | Heterogeneous nuclear ribonucleoprotein H3 | 0.33 | 0.15 |
|  | EIF4A3 | P38919 | 46.871 | Eukaryotic initiation factor 4A-III | 0.32 | 0.00 |
|  | C1QBP | Q07021 | 31.362 | Complement component 1 Q subcomponent-binding protein, mitochondrial | 0.32 | 0.00 |
|  | MIB1 | Q86YT6 | 110.136 | E3 ubiquitin-protein ligase MIB1 | 0.30 | 0.00 |
|  | ATAD3B | Q5T9A4 | 72.573 | ATPase family AAA domain-containing protein 3B | 0.27 | 0.00 |
|  | LBR | Q14739 | 70.703 | Lamin-B receptor | 0.21 | 0.00 |
|  | SF3B3 | Q15393 | 135.577 | Splicing factor 3B subunit 3 | 0.19 | 0.02 |
|  | MTHFD1L | P11586 | 105.79 | Monofunctional C1-tetrahydrofolate synthase, mitochondrial | 0.18 | 0.00 |
|  | RPL3 | P39023 | 46.109 | 60S ribosomal protein L3 | 0.18 | 0.00 |
|  | TCP1 | P17987 | 60.344 | T-complex protein 1 subunit alpha | 0.17 | 0.00 |
|  | RCC1 | P18754 | 44.969 | Regulator of chromosome condensation | 0.14 | 0.00 |
|  | PRPF31 | Q8WWY3 | 55.456 | U4/U6 small nuclear ribonucleoprotein Prp31 | 0.14 | 0.00 |
|  | LAS1L | Q9Y4W2 | 83.065 | Ribosomal biogenesis protein LAS1L | 0.12 | 0.00 |
|  | SNRNP200 | O75643 | 244.508 | U5 small nuclear ribonucleoprotein 200 kDa helicase | 0.12 | 0.00 |
|  | NAT10 | Q9H0A0 | 115.73 | RNA cytidine acetyltransferase | 0.10 | 0.00 |
|  | AMOT | Q4VCS5 | 118.09 | Angiomotin | 0.09 | 0.00 |
|  | MYO1C | O00159 | 121.682 | Unconventional myosin-Ic | 0.09 | 0.00 |
|  | EFTUD2 | Q15029 | 109.436 | 116 kDa U5 small nuclear ribonucleoprotein component | 0.08 | 0.00 |
|  | DSG2 | Q14126 | 122.294 | Desmoglein-2 | 0.07 | 0.00 |
|  | SMARCA5 | O60264 | 121.905 | SWI/SNF-related matrix-associated actin-dependent regulator of chromatin subfamily A member 5 | 0.06 | 0.00 |
|  | PRPF8 | Q6P2Q9 | 273.6 | Pre-mRNA-processing-splicing factor 8 | 0.05 | 0.00 |
|  | DHX30 | Q7L2E3 | 133.938 | Putative ATP-dependent RNA helicase DHX30 | 0.05 | 0.00 |
|  | NUMA1 | Q14980 | 238.26 | Nuclear mitotic apparatus protein 1 | 0.05 | 0.00 |
|  | MDN1 | Q9NU22 | 632.82 | Midasin | 0.04 | 0.00 |

Supplementary Table 4. Functional enrichment analysis of ANKRD55 interactome from total protein extracts. The algorithm DAVID was used to analyze the ANKRD55 interactome. The GO and other terms with p value<0.001 are shown and grouped by enrichment score.

| TOTAL PROTEIN EXTRACTS | Annotation Cluster 1 - Enrichment Score: 12.73 | |  |  |  |  |  |  |  |  |
| --- | --- | --- | --- | --- | --- | --- | --- | --- | --- | --- |
|  | **Category** | **Term** | **Count** | **%** | **PValue** | **Genes** | **Fold Enrichment** | **Bonferroni** | **Benjamini** | **FDR** |
|  | UP_KEYWORDS | Nucleotide-binding | 52 | 34,90 | 1,26E-18 | P17812, Q9Y5M8, O43615, O00571, Q5T9A4, Q15029, P10398, P18085, Q9Y3I0, P53396, Q00535, P05023, Q9BUF5, Q14566, Q9Y285, Q6P1M0, Q5T160, P49588, P41252, P50570, P28288, P38646, Q8NI60, P42356, Q9NU22, Q9P2J5, Q9NSD9, Q14683, P56192, Q9BSD7, P45880, Q6UB35, P54136, P16615, P78527, P27708, P40616, P17980, P50990, P50991, P38919, Q92900, Q9NR30, O75643, P54886, P51148, P25205, P33993, P26640, Q13085, Q14204, Q15645 | 4,02 | 2,93E-16 | 9,75E-17 | 1,61E-15 |
|  | UP_KEYWORDS | ATP-binding | 43 | 28,86 | 5,74E-16 | P17812, P56192, O43615, Q9BSD7, Q5T9A4, O00571, P10398, Q6UB35, P54136, P16615, P78527, P27708, Q9Y3I0, P17980, P53396, Q00535, P50990, P50991, P38919, P05023, Q92900, Q14566, Q9Y285, Q9NR30, Q5T160, P49588, O75643, P41252, P54886, P28288, P38646, P25205, P33993, P26640, Q13085, Q8NI60, P42356, Q14204, Q14683, Q9NSD9, 9P2J5, Q9NU22, Q15645 | 4,27 | 1,29E-13 | 3,23E-14 | 7,11E-13 |
|  | GOTERM_MF_DIRECT | GO:0005524~ATP binding | 45 | 30,20 | 1,18E-13 | P17812, P31689, P56192, O43615, Q9BSD7, Q5T9A4, O00571, P10398, Q6UB35, P54136, P16615, P78527, P27708, Q9Y3I0, P17980, P53396, Q00535, P50990, P50991, P38919, P05023, Q92900, Q14566, Q9Y285, Q9NR30, Q5T160, P49588, O75643, P41252, O60884, P54886, P28288, P38646, P25205, P33993, P26640, Q13085, Q8NI60, P42356, Q14204, Q14683, Q9NSD9, Q9P2J5, Q9NU22, Q15645 | 3,48 | 4,39E-11 | 2,19E-11 | 1,62E-10 |
|  | **Annotation Cluster 2 - Enrichment Score: 8.13** | |  |  |  |  |  |  |  |  |
|  | **Category** | **Term** | **Count** | **%** | **PValue** | **Genes** | **Fold Enrichment** | **Bonferroni** | **Benjamini** | **FDR** |
|  | GOTERM_BP_DIRECT | GO:0006412~translation | 20 | 13,42 | 6,31E-13 | P15880, P36578, P53007, P05388, Q92616, P27635, Q15029, P32969, P05141, Q00325, P62424, O75746, Q9NSD9, P61247, P62829, Q02978, P62244, P23396, Q9UJS0, P46781 | 9,09 | 6,09E-10 | 6,09E-10 | 9,93E-10 |
|  | GOTERM_BP_DIRECT | GO:0000184~nuclear-transcribed mRNA catabolic process, nonsense-mediated decay | 15 | 10,07 | 1,85E-12 | P15880, P36578, P63151, P05388, P30153, P27635, P32969, P62424, P61247, P62829, P38919, P62244, P23396, Q92900, P46781 | 14,50 | 1,78E-09 | 8,90E-10 | 2,91E-09 |
|  | GOTERM_CC_DIRECT | GO:0005925~focal adhesion | 21 | 14,09 | 5,15E-11 | P15880, P36578, Q9Y490, P35613, P05388, P50570, P21333, P31946, P23528, P61981, P38646, P32969, P42356, P62258, P62424, P61247, P62829, Q00610, P23396, Q15366, P46781 | 6,61 | 1,31E-08 | 2,61E-09 | 6,70E-08 |
|  | GOTERM_MF_DIRECT | GO:0003735~structural constituent of ribosome | 17 | 11,41 | 8,05E-11 | P15880, P36578, P53007, P05388, P27635, P32969, P05141, Q00325, P62424, O75746, P61247, P62829, Q02978, P62244, P23396, Q9UJS0, P46781 | 8,85 | 3,00E-08 | 7,51E-09 | 1,11E-07 |
|  | GOTERM_BP_DIRECT | GO:0019083~viral transcription | 13 | 8,72 | 2,29E-10 | Q12769, P15880, P36578, Q92621, P62424, P05388, P61247, P62829, P27635, P62244, P23396, P46781, P32969 | 13,35 | 2,21E-07 | 7,36E-08 | 3,60E-07 |
|  | UP_KEYWORDS | Ribonucleoprotein | 17 | 11,41 | 4,44E-10 | P15880, P36578, O75643, P05388, P27635, Q15029, O00567, P32969, P62424, P61247, P62829, Q6P2Q9, P62244, P23396, Q15366, Q15365, P46781 | 7,93 | 1,04E-07 | 1,73E-08 | 5,71E-07 |
|  | GOTERM_BP_DIRECT | GO:0006614~SRP-dependent cotranslational protein targeting to membrane | 12 | 8,05 | 5,08E-10 | Q9Y5M8, P15880, P36578, P62424, P05388, P61247, P62829, P27635, P62244, P23396, P46781, P32969 | 14,68 | 4,90E-07 | 1,22E-07 | 7,99E-07 |
|  | GOTERM_BP_DIRECT | GO:0006364~rRNA processing | 15 | 10,07 | 4,88E-09 | P15880, Q9NR30, P36578, P05388, P27635, O00567, P32969, P62424, Q9NU22, P61247, P62829, P38919, P62244, P23396, P46781 | 8,06 | 4,71E-06 | 7,85E-07 | 7,68E-06 |
|  | GOTERM_BP_DIRECT | GO:0006413~translational initiation | 12 | 8,05 | 2,84E-08 | P15880, P36578, P62424, P05388, P61247, Q14152, P62829, P27635, P62244, P23396, P46781, P32969 | 10,07 | 2,74E-05 | 3,91E-06 | 4,47E-05 |
|  | GOTERM_CC_DIRECT | GO:0005840~ribosome | 12 | 8,05 | 1,05E-07 | P15880, P36578, P62424, P05388, Q92616, P61247, P62829, P27635, P62244, P23396, P46781, P32969 | 8,90 | 2,66E-05 | 2,05E-06 | 1,36E-04 |
|  | UP_KEYWORDS | Ribosomal protein | 11 | 7,38 | 9,41E-07 | P15880, P36578, P62424, P05388, P61247, P62829, P27635, P62244, P23396, P46781, P32969 | 8,21 | 2,19E-04 | 1,57E-05 | 1,21E-03 |
|  | **Annotation Cluster 3 - Enrichment Score: 6.987** | |  |  |  |  |  |  |  |  |
|  | **Category** | **Term** | **Count** | **%** | **PValue** | **Genes** | **Fold Enrichment** | **Bonferroni** | **Benjamini** | **FDR** |
|  | GOTERM_MF_DIRECT | GO:0098641~cadherin binding involved in cell-cell adhesion | 16 | 10,74 | 3,00E-08 | P15880, Q9Y490, P35613, Q92616, O00571, P49327, P21333, P31946, O00567, P54136, P42356, P62258, P62424, P63244, P50990, Q15365 | 6,38 | 1,12E-05 | 1,87E-06 | 4,14E-05 |
|  | GOTERM_CC_DIRECT | GO:0005913~cell-cell adherens junction | 16 | 10,74 | 5,55E-08 | P15880, Q9Y490, P35613, Q92616, O00571, P49327, P21333, P31946, O00567, P54136, P42356, P62258, P62424, P63244, P50990, Q15365 | 6,10 | 1,41E-05 | 1,28E-06 | 7,23E-05 |
|  | GOTERM_BP_DIRECT | GO:0098609~cell-cell adhesion | 14 | 9,40 | 6,56E-07 | P15880, P35613, Q92616, O00571, P49327, P31946, O00567, P54136, P42356, P62258, P62424, P63244, P50990, Q15365 | 5,94 | 6,33E-04 | 7,91E-05 | 1,03E-03 |
|  | **Annotation Cluster 4 - Enrichment Score: 6.97** | |  |  |  |  |  |  |  |  |
|  | **Category** | **Term** | **Count** | **%** | **PValue** | **Genes** | **Fold Enrichment** | **Bonferroni** | **Benjamini** | **FDR** |
|  | UP_KEYWORDS | Mitochondrion | 28 | 18,79 | 2,69E-08 | P31689, P53007, O43615, Q3ZCQ8, P45880, Q07021, O00571, Q5T9A4, Q6UB35, O95831, P05141, O75746, P13804, Q02978, P36542, P23396, Q9UJS0, Q96HS1, Q5T160, P54886, P04181, P38646, P11177, Q8NI60, P42704, Q9H9B4, P48047, Q00325 | 3,46 | 6,26E-06 | 8,95E-07 | 3,45E-05 |
|  | UP_KEYWORDS | Mitochondrion inner membrane | 14 | 9,40 | 8,07E-08 | P53007, O43615, Q3ZCQ8, Q5T9A4, P54886, O95831, P05141, P48047, Q00325, O75746, Q02978, P36542, P23396, Q9UJS0 | 7,16 | 1,88E-05 | 1,88E-06 | 1,04E-04 |
|  | GOTERM_CC_DIRECT | GO:0005743~mitochondrial inner membrane | 17 | 11,41 | 5,65E-07 | P53007, O43615, Q3ZCQ8, P45880, Q5T9A4, P28288, P54886, O95831, P05141, Q9H9B4, P48047, Q00325, O75746, Q02978, P36542, P23396, Q9UJS0 | 4,75 | 1,43E-04 | 1,02E-05 | 7,36E-04 |
|  | **Annotation Cluster 5 - Enrichment Score: 5.30** | |  |  |  |  |  |  |  |  |
|  | **Category** | **Term** | **Count** | **%** | **PValue** | **Genes** | **Fold Enrichment** | **Bonferroni** | **Benjamini** | **FDR** |
|  | INTERPRO | IPR016024:Armadillo-type fold | 19 | 12,75 | 3,28E-10 | Q96T76, P55060, Q92616, Q8TEX9, P30153, Q9UBB4, Q93008, Q86VP6, P78527, P42695, Q9Y678, P42356, O14980, P52292, Q9BQG0, P50851, Q00610, O43592, O00410 | 6,83 | 1,57E-07 | 1,57E-07 | 4,70E-07 |
|  | UP_SEQ_FEATURE | repeat:HEAT 2 | 10 | 6,71 | 3,96E-10 | Q96T76, P42695, Q9Y678, O14980, Q92616, Q8TEX9, P30153, Q86VP6, P78527, O00410 | 23,22 | 2,37E-07 | 2,37E-07 | 5,85E-07 |
|  | UP_SEQ_FEATURE | repeat:HEAT 1 | 10 | 6,71 | 3,96E-10 | Q96T76, P42695, Q9Y678, O14980, Q92616, Q8TEX9, P30153, Q86VP6, P78527, O00410 | 23,22 | 2,37E-07 | 2,37E-07 | 5,85E-07 |
|  | UP_SEQ_FEATURE | repeat:HEAT 4 | 9 | 6,04 | 8,45E-10 | Q96T76, P42695, Q9Y678, O14980, Q92616, Q8TEX9, P30153, Q86VP6, O00410 | 28,18 | 5,06E-07 | 2,53E-07 | 1,25E-06 |
|  | UP_SEQ_FEATURE | repeat:HEAT 3 | 9 | 6,04 | 2,99E-09 | Q96T76, P42695, Q9Y678, O14980, Q92616, Q8TEX9, P30153, Q86VP6, O00410 | 24,24 | 1,79E-06 | 5,98E-07 | 4,42E-06 |
|  | INTERPRO | IPR011989:Armadillo-like helical | 14 | 9,40 | 2,65E-08 | Q96T76, P55060, Q92616, Q8TEX9, P30153, Q9UBB4, Q86VP6, P78527, P42695, Q9Y678, O14980, P52292, O43592, O00410 | 7,86 | 1,27E-05 | 6,33E-06 | 3,79E-05 |
|  | UP_SEQ_FEATURE | repeat:HEAT 6 | 7 | 4,70 | 7,47E-08 | Q96T76, O14980, Q92616, Q8TEX9, P30153, Q86VP6, O00410 | 31,42 | 4,47E-05 | 1,12E-05 | 1,10E-04 |
|  | UP_SEQ_FEATURE | repeat:HEAT 5 | 7 | 4,70 | 1,98E-07 | Q96T76, O14980, Q92616, Q8TEX9, P30153, Q86VP6, O00410 | 26,93 | 1,19E-04 | 1,98E-05 | 2,92E-04 |
|  | **Annotation Cluster 6 - Enrichment Score: 5.25** | |  |  |  |  |  |  |  |  |
|  | **Category** | **Term** | **Count** | **%** | **PValue** | **Genes** | **Fold Enrichment** | **Bonferroni** | **Benjamini** | **FDR** |
|  | INTERPRO | IPR027417:P-loop containing nucleoside triphosphate hydrolase | 24 | 16,11 | 3,77E-07 | P17812, Q9Y5M8, Q9NR30, O75643, Q9BSD7, P50570, O00571, Q5T9A4, Q15029, P28288, Q6UB35, P51148, P25205, P33993, P18085, Q14204, Q14683, Q9NU22, P40616, P17980, Q15645, P38919, Q92900, Q14566 | 3,45 | 1,80E-04 | 3,60E-05 | 5,39E-04 |
|  | **Annotation Cluster 7 - Enrichment Score: 5.25** | |  |  |  |  |  |  |  |  |
|  | **Category** | **Term** | **Count** | **%** | **PValue** | **Genes** | **Fold Enrichment** | **Bonferroni** | **Benjamini** | **FDR** |
|  | UP_KEYWORDS | Aminoacyl-tRNA synthetase | 9 | 6,04 | 2,41E-10 | P26640, Q9Y285, P56192, Q5T160, P49588, Q9P2J5, Q9NSD9, P41252, P54136 | 32,71 | 5,61E-08 | 1,12E-08 | 3,09E-07 |
|  | GOTERM_BP_DIRECT | GO:0006418~tRNA aminoacylation for protein translation | 9 | 6,04 | 1,55E-09 | P26640, Q9Y285, P56192, Q5T160, P49588, Q9P2J5, Q9NSD9, P41252, P54136 | 25,88 | 1,49E-06 | 2,99E-07 | 2,44E-06 |
|  | UP_KEYWORDS | Ligase | 16 | 10,74 | 3,24E-08 | P17812, Q9Y285, Q86YT6, P56192, Q6P1M0, Q5T160, P49588, P41252, P54136, Q6UB35, P26640, Q13085, P27708, Q9Y3I0, Q9P2J5, Q9NSD9 | 6,37 | 7,56E-06 | 8,39E-07 | 4,17E-05 |
|  | INTERPRO | IPR009080:Aminoacyl-tRNA synthetase, class 1a, anticodon-binding | 6 | 4,03 | 3,44E-08 | P26640, P56192, Q5T160, Q9P2J5, P41252, P54136 | 58,27 | 1,64E-05 | 5,48E-06 | 4,92E-05 |
|  | INTERPRO | IPR001412:Aminoacyl-tRNA synthetase, class I, conserved site | 6 | 4,03 | 7,93E-08 | P26640, P56192, Q5T160, Q9P2J5, P41252, P54136 | 50,50 | 3,79E-05 | 9,47E-06 | 1,13E-04 |
|  | UP_SEQ_FEATURE | short sequence motif:"HIGH" region | 6 | 4,03 | 1,62E-07 | P26640, P56192, Q5T160, Q9P2J5, P41252, P54136 | 44,88 | 9,70E-05 | 1,94E-05 | 2,39E-04 |
|  | INTERPRO | IPR014729:Rossmann-like alpha/beta/alpha sandwich fold | 7 | 4,70 | 8,88E-07 | P26640, P56192, Q5T160, Q9P2J5, P41252, P13804, P54136 | 21,04 | 4,24E-04 | 7,08E-05 | 1,27E-03 |
|  | UP_KEYWORDS | Protein biosynthesis | 10 | 6,71 | 1,58E-06 | P26640, Q9Y285, P56192, Q5T160, P49588, Q9P2J5, Q9NSD9, P41252, Q14152, P54136 | 9,09 | 3,67E-04 | 2,30E-05 | 2,03E-03 |
|  | KEGG_PATHWAY | hsa00970:Aminoacyl-tRNA biosynthesis | 9 | 6,04 | 6,46E-06 | P26640, Q9Y285, P56192, Q5T160, P49588, Q9P2J5, Q9NSD9, P41252, P54136 | 8,81 | 9,82E-04 | 9,82E-04 | 7,74E-03 |
|  | **Annotation Cluster 8 - Enrichment Score: 4.95** | |  |  |  |  |  |  |  |  |
|  | **Category** | **Term** | **Count** | **%** | **PValue** | **Genes** | **Fold Enrichment** | **Bonferroni** | **Benjamini** | **FDR** |
|  | UP_KEYWORDS | Mitochondrion | 28 | 18,79 | 2,69E-08 | P31689, P53007, O43615, Q3ZCQ8, P45880, Q07021, O00571, Q5T9A4, Q6UB35, O95831, P05141, O75746, P13804, Q02978, P36542, P23396, Q9UJS0, Q96HS1, Q5T160, P54886, P04181, P38646, P11177, Q8NI60, P42704, Q9H9B4, P48047, Q00325 | 3,46 | 6,26E-06 | 8,95E-07 | 3,45E-05 |
|  | UP_KEYWORDS | Transit peptide | 16 | 10,74 | 7,73E-06 | Q5T160, P53007, O43615, Q3ZCQ8, Q07021, Q6UB35, P04181, P38646, O95831, P11177, Q8NI60, P42704, P48047, Q00325, P13804, P36542 | 4,12 | 1,80E-03 | 1,00E-04 | 9,93E-03 |
|  | **Annotation Cluster 9 - Enrichment Score: 3.84** | |  |  |  |  |  |  |  |  |
|  | **Category** | **Term** | **Count** | **%** | **PValue** | **Genes** | **Fold Enrichment** | **Bonferroni** | **Benjamini** | **FDR** |
|  | UP_KEYWORDS | Neurodegeneration | 13 | 8,72 | 1,44E-06 | P56192, Q5T160, P49588, P50570, P54886, O00567, Q9UBB4, O95831, Q8NI60, Q14204, Q8IY17, Q00535, P12004 | 6,13 | 3,36E-04 | 2,24E-05 | 1,85E-03 |
|  | **Annotation Cluster 10 - Enrichment Score: 3.44** | |  |  |  |  |  |  |  |  |
|  | **Category** | **Term** | **Count** | **%** | **PValue** | **Genes** | **Fold Enrichment** | **Bonferroni** | **Benjamini** | **FDR** |
|  | UP_KEYWORDS | Mitochondrion inner membrane | 14 | 9,40 | 8,07E-08 | P53007, O43615, Q3ZCQ8, Q5T9A4, P54886, O95831, P05141, P48047, Q00325, O75746, Q02978, P36542, P23396, Q9UJS0 | 7,16 | 1,88E-05 | 1,88E-06 | 1,04E-04 |

Supplementary Table 5. Prediction of binding ability of ANKRD55 residues to DNA, RNA, ATP, ADP, AMP, GTP and GDP. The first and second columns display the ANKRD55 isoform 001 protein sequence and amino acid number, which are followed by the positive results of predictions for 7 ligand types: DNA, RNA, ATP, ADP, AMP, GTP, and GDP. Predictions for each ligand type include two columns that provide annotation of predicted binding (each ligand is annotated as either binding (B or 1) or non-binding (N or 0)) and prediction scores which estimate probability of binding to a given ligand. Residues with the probability > 0.4727 for the prediction of DNA-binding and > 0.1493 for the prediction of RNA-binding are annotated as putative DNA- and RNA-binding residues, respectively.

| **Amino Acid number** | **Amino Acid** | **Binary DNA** | **DNA prob** | **Binary RNA** | **RNA prob** | **ATP binding** | **ATP prob** | **ADP binding** | **ADP prob** | **AMP binding** | **AMP prob** | **GTP binding** | **GTP prob** | **GDP binding** | **GDP prob** |
| --- | --- | --- | --- | --- | --- | --- | --- | --- | --- | --- | --- | --- | --- | --- | --- |
| 10 | S | **1** | **0.5018** | 0 | 0.042 | N | 0.023 | N | 0.027 | N | 0.033 | N | 0.011 | N | 0.019 |
| 29 | T | 0 | 0.1676 | 0 | 0.0583 | N | 0.035 | N | 0.024 | N | 0.034 | **B** | **0.077** | N | 0.021 |
| 36 | S | 0 | 0.2208 | 0 | 0.0504 | **B** | **0.135** | N | 0.027 | N | 0.027 | N | 0.026 | N | 0.021 |
| 37 | N | 0 | 0.336 | 0 | 0.0507 | **B** | **0.129** | N | 0.027 | N | 0.023 | N | 0.028 | N | 0.018 |
| 64 | P | 0 | 0.1264 | 0 | 0.051 | N | 0.061 | **B** | **0.153** | N | 0.036 | N | 0.053 | N | 0.028 |
| 71 | G | 0 | 0.3358 | 0 | 0.0404 | **B** | **0.11** | N | 0.025 | N | 0.024 | N | 0.031 | N | 0.018 |
| 72 | R | **1** | **0.538** | 0 | 0.0407 | N | 0.029 | N | 0.024 | N | 0.035 | N | 0.027 | N | 0.021 |
| 73 | Q | **1** | **0.4985** | 0 | 0.0408 | N | 0.029 | N | 0.029 | N | 0.029 | N | 0.026 | N | 0.018 |
| 93 | Y | **1** | **0.5458** | 0 | 0.0392 | N | 0.03 | N | 0.039 | N | 0.027 | N | 0.024 | N | 0.016 |
| 97 | S | 0 | 0.1567 | 0 | 0.0383 | N | 0.07 | **B** | **0.162** | N | 0.035 | N | 0.052 | N | 0.028 |
| 99 | C | 0 | 0.2287 | 0 | 0.0398 | N | 0.088 | N | 0.048 | N | 0.038 | **B** | **0.075** | N | 0.033 |
| 103 | Y | 0 | 0.2464 | 0 | 0.0449 | **B** | **0.1** | N | 0.028 | N | 0.027 | N | 0.027 | N | 0.02 |
| 119 | K | **1** | **0.5568** | 0 | 0.0428 | N | 0.026 | N | 0.023 | N | 0.03 | N | 0.032 | N | 0.021 |
| 130 | P | 0 | 0.1219 | 0 | 0.047 | N | 0.054 | **B** | **0.239** | N | 0.034 | N | 0.063 | N | 0.028 |
| 136 | A | 0 | 0.1375 | 0 | 0.0553 | **B** | **0.185** | N | 0.029 | N | 0.027 | N | 0.029 | N | 0.021 |
| 137 | E | 0 | 0.1561 | 0 | 0.0624 | **B** | **0.189** | N | 0.027 | N | 0.023 | N | 0.031 | N | 0.019 |
| 165 | P | 0 | 0.1058 | 0 | 0.0488 | N | 0.059 | **B** | **0.275** | N | 0.034 | N | 0.073 | N | 0.029 |
| 171 | F | 0 | 0.1937 | 0 | 0.0494 | **B** | **0.153** | N | 0.03 | N | 0.029 | N | 0.028 | N | 0.021 |
| 172 | H | 0 | 0.3408 | 0 | 0.0518 | **B** | **0.127** | N | 0.027 | N | 0.025 | N | 0.03 | N | 0.018 |
| 198 | A | 0 | 0.1158 | 0 | 0.0523 | N | 0.058 | **B** | **0.251** | N | 0.036 | N | 0.06 | N | 0.03 |
| 200 | H | 0 | 0.2455 | 0 | 0.0555 | N | 0.052 | N | 0.053 | N | 0.046 | **B** | **0.075** | N | 0.031 |
| 204 | Q | 0 | 0.2954 | 0 | 0.0557 | **B** | **0.162** | N | 0.029 | N | 0.027 | N | 0.028 | N | 0.021 |
| 205 | S | 0 | 0.3059 | 0 | 0.0559 | **B** | **0.142** | N | 0.029 | N | 0.027 | N | 0.027 | N | 0.019 |
| 234 | C | 0 | 0.1443 | 0 | 0.0486 | N | 0.047 | **B** | **0.266** | N | 0.036 | N | 0.072 | N | 0.029 |
| 240 | A | 0 | 0.26 | 0 | 0.0507 | **B** | **0.202** | N | 0.031 | N | 0.028 | N | 0.027 | N | 0.021 |
| 241 | A | 0 | 0.2557 | 0 | 0.0556 | **B** | **0.169** | N | 0.028 | N | 0.024 | N | 0.029 | N | 0.019 |
| 268 | P | 0 | 0.1162 | 0 | 0.0558 | N | 0.054 | **B** | **0.21** | N | 0.035 | N | 0.059 | N | 0.028 |
| 274 | A | 0 | 0.1587 | 0 | 0.0538 | **B** | **0.145** | N | 0.029 | N | 0.026 | N | 0.027 | N | 0.021 |
| 275 | A | 0 | 0.1808 | 0 | 0.0541 | **B** | **0.147** | N | 0.025 | N | 0.025 | N | 0.032 | N | 0.018 |
| 301 | P | 0 | 0.134 | 0 | 0.0465 | N | 0.057 | **B** | **0.204** | N | 0.034 | N | 0.052 | N | 0.028 |
| 303 | A | 0 | 0.1777 | 0 | 0.0477 | N | 0.08 | N | 0.052 | N | 0.039 | **B** | **0.076** | N | 0.029 |
| 307 | Y | 0 | 0.3624 | 0 | 0.0479 | **B** | **0.125** | N | 0.03 | N | 0.028 | N | 0.031 | N | 0.021 |
| 308 | C | 0 | 0.2689 | 0 | 0.0485 | **B** | **0.123** | N | 0.027 | N | 0.027 | N | 0.03 | N | 0.019 |
| 409 | K | **1** | **0.5325** | 0 | 0.0423 | N | 0.032 | N | 0.023 | N | 0.049 | N | 0.025 | N | 0.02 |
| 433 | R | **1** | **0.4811** | 0 | 0.0539 | N | 0.024 | N | 0.021 | N | 0.022 | N | 0.028 | N | 0.011 |
| 444 | N | **1** | **0.5579** | 0 | 0.0353 | N | 0.038 | N | 0.025 | N | 0.028 | N | 0.018 | N | 0.024 |
| 448 | T | **1** | 0.5703 | 0 | 0.0321 | N | 0.034 | N | 0.025 | N | 0.038 | N | 0.053 | N | 0.026 |
| 449 | A | 0 | 0.3052 | 0 | 0.0289 | N | 0.055 | **B** | **0.161** | N | 0.04 | N | 0.042 | N | 0.026 |
| 450 | S | **1** | **0.7072** | 0 | 0.0317 | N | 0.059 | N | 0.056 | N | 0.041 | N | 0.032 | N | 0.031 |
| 451 | H | **1** | **0.7067** | 0 | 0.0325 | **B** | **0.112** | N | 0.088 | N | 0.037 | N | 0.066 | N | 0.038 |
| 452 | R | **1** | **0.7105** | 0 | 0.032 | **B** | **0.12** | N | 0.024 | N | 0.025 | N | 0.014 | N | 0.023 |
| 454 | T | **1** | 0.6075 | 0 | 0.0318 | N | 0.038 | N | 0.025 | N | 0.03 | N | 0.021 | N | 0.015 |
| 455 | S | **1** | **0.5098** | 0 | 0.0329 | N | 0.065 | N | 0.029 | N | 0.026 | N | 0.023 | N | 0.017 |
| 456 | H | **1** | **0.726** | 0 | 0.0335 | **B** | **0.104** | N | 0.023 | N | 0.021 | N | 0.028 | N | 0.017 |
| 460 | S | **1** | **0.5036** | 0 | 0.0331 | N | 0.018 | N | 0.016 | N | 0.026 | N | 0.018 | N | 0.013 |
| 461 | S | **1** | **0.4933** | 0 | 0.0334 | N | 0.026 | N | 0.024 | N | 0.035 | N | 0.018 | N | 0.014 |
| 465 | H | **1** | **0.6196** | 0 | 0.042 | N | 0.021 | N | 0.02 | N | 0.024 | N | 0.022 | N | 0.012 |
| 469 | R | **1** | **0.6162** | 0 | 0.0341 | N | 0.035 | N | 0.027 | N | 0.019 | N | 0.023 | N | 0.016 |
| 470 | S | **1** | **0.5977** | 0 | 0.0345 | N | 0.031 | N | 0.017 | N | 0.025 | N | 0.025 | N | 0.015 |
| 471 | Q | **1** | **0.6657** | 0 | 0.0357 | N | 0.023 | N | 0.02 | N | 0.026 | N | 0.024 | N | 0.019 |
| 472 | K | **1** | **0.6683** | 0 | 0.036 | N | 0.025 | N | 0.028 | N | 0.03 | N | 0.021 | N | 0.019 |
| 473 | S | **1** | **0.6649** | 0 | 0.0355 | N | 0.022 | N | 0.023 | N | 0.028 | N | 0.021 | N | 0.019 |
| 474 | R | **1** | **0.6931** | 0 | 0.0349 | N | 0.025 | N | 0.028 | N | 0.031 | N | 0.014 | N | 0.02 |
| 483 | R | **1** | **0.5902** | 0 | 0.0399 | N | 0.026 | N | 0.015 | N | 0.045 | N | 0.012 | N | 0.011 |
| 495 | K | **1** | **0.6468** | 0 | 0.0357 | N | 0.026 | N | 0.043 | N | 0.02 | N | 0.017 | N | 0.013 |
| 499 | N | **1** | **0.5911** | 0 | 0.0341 | N | 0.015 | N | 0.017 | N | 0.017 | N | 0.023 | N | 0.041 |
| 500 | Q | **1** | **0.5134** | 0 | 0.0341 | N | 0.017 | N | 0.014 | N | 0.021 | N | 0.02 | N | 0.025 |
| 505 | K | **1** | **0.5171** | 0 | 0.037 | N | 0.026 | N | 0.03 | N | 0.031 | N | 0.023 | N | 0.025 |
| 507 | W | **1** | **0.5383** | 0 | 0.0373 | N | 0.013 | N | 0.027 | N | 0.029 | N | 0.025 | N | 0.016 |
| 508 | T | **1** | **0.5095** | 0 | 0.0384 | N | 0.025 | N | 0.022 | N | 0.034 | N | 0.017 | N | 0.016 |
| 514 | K | **1** | **0.4995** | 0 | 0.0413 | N | 0.025 | N | 0.027 | N | 0.031 | N | 0.016 | N | 0.012 |
| 523 | R | **1** | **0.4825** | 0 | 0.0429 | N | 0.027 | N | 0.02 | N | 0.035 | N | 0.018 | N | 0.012 |
| 540 | N | **1** | **0.561** | 0 | 0.0436 | N | 0.02 | N | 0.031 | N | 0.018 | N | 0.01 | N | 0.029 |
| 545 | Q | **1** | **0.516** | 0 | 0.0397 | N | 0.022 | N | 0.026 | N | 0.017 | N | 0.02 | N | 0.007 |
| 546 | N | **1** | **0.5599** | 0 | 0.0394 | N | 0.015 | N | 0.019 | N | 0.016 | N | 0.005 | N | 0.005 |
| 548 | Q | **1** | **0.5873** | 0 | 0.0429 | N | 0.011 | N | 0.012 | N | 0.014 | N | 0.014 | N | 0.02 |
| 554 | R | **1** | **0.4948** | 0 | 0.0387 | N | 0.023 | N | 0.028 | N | 0.054 | N | 0.016 | N | 0.036 |
| 556 | K | **1** | **0.4759** | 0 | 0.0486 | N | 0.032 | N | 0.026 | N | 0.021 | N | 0.033 | N | 0.011 |
| 564 | R | **1** | **0.5806** | 0 | 0.0415 | N | 0.029 | N | 0.023 | N | 0.033 | N | 0.012 | N | 0.01 |
| 565 | N | **1** | **0.5489** | 0 | 0.0413 | N | 0.036 | N | 0.054 | N | 0.02 | N | 0.02 | N | 0.019 |
| 566 | N | **1** | **0.5078** | 0 | 0.0415 | N | 0.03 | N | 0.017 | N | 0.025 | N | 0.036 | N | 0.011 |
| 569 | P | 0 | 0.1592 | 0 | 0.041 | N | 0.01 | N | 0.007 | N | 0.055 | **B** | **0.098** | N | 0.017 |
| 582 | R | **1** | **0.5988** | 0 | 0.0377 | N | 0.053 | N | 0.028 | N | 0.028 | N | 0.012 | N | 0.015 |
| 585 | R | **1** | **0.575** | 0 | 0.0385 | N | 0.029 | N | 0.018 | N | 0.017 | N | 0.011 | N | 0.01 |
| 592 | S | **1** | **0.475** | 0 | 0.0342 | N | 0.03 | N | 0.033 | N | 0.023 | N | 0.017 | N | 0.017 |
| 593 | Q | **1** | **0.5741** | 0 | 0.034 | N | 0.036 | N | 0.048 | N | 0.027 | N | 0.02 | N | 0.021 |
| 594 | R | **1** | **0.7255** | 0 | 0.0331 | N | 0.056 | N | 0.016 | N | 0.03 | N | 0.025 | N | 0.027 |
| 595 | R | **1** | **0.6229** | 0 | 0.0331 | N | 0.021 | N | 0.025 | N | 0.017 | N | 0.021 | N | 0.015 |
| 596 | H | **1** | **0.644** | 0 | 0.0342 | N | 0.024 | N | 0.02 | N | 0.025 | N | 0.019 | N | 0.008 |

Supplementary Table 6. IPA canonical signaling pathways enrichment in the ANKRD55 interactome from nuclear and total protein extract. Significantly enriched pathways (-Log (p-value)>1.30) are shown.

|  | Ingenuity Canonical Pathways | -Log(p-value) | Molecules |
| --- | --- | --- | --- |
| TOTAL PROTEIN EXTRACTS | tRNA Charging | 11,40 | LARS,RARS,RARS2,AARS,VARS,MARS,IARS,FARSA,FARSB |
|  | EIF2 Signaling | 9,49 | PTBP1,RPL4,RPS3A,EIF4A3,RPS9,EIF3A,RPL10,RPS15A,RPL23,RPS2,RPS3,RPL7A,RPL9,RPLP0 |
|  | Regulation of eIF4 and p70S6K Signaling | 5,91 | PPP2R1A,RPS3A,PPP2R2A,EIF4A3,RPS9,EIF3A,RPS15A,RPS2,RPS3 |
|  | RAN Signaling | 5,36 | CSE1L,XPO1,KPNA2,IPO5 |
|  | mTOR Signaling | 5,03 | PPP2R1A,RPS3A,PPP2R2A,EIF4A3,RPS9,EIF3A,RPS15A,RPS2,RPS3 |
|  | Cell Cycle: G2/M DNA Damage Checkpoint Regulation | 4,66 | PRKDC,YWHAG,YWHAH,YWHAE,YWHAB |
|  | HIPPO signaling | 4,56 | PPP2R1A,YWHAG,YWHAH,YWHAE,YWHAB,PPP2R2A |
|  | Cell Cycle Control of Chromosomal Replication | 4,42 | MCM3,MCM6,PCNA,CDK5,MCM7 |
|  | PI3K/AKT Signaling | 3,69 | PPP2R1A,YWHAG,YWHAH,YWHAE,YWHAB,PPP2R2A |
|  | Proline Biosynthesis I | 3,57 | PYCR3,ALDH18A1 |
|  | p70S6K Signaling | 3,56 | PPP2R1A,YWHAG,YWHAH,YWHAE,YWHAB,PPP2R2A |
|  | ERK/MAPK Signaling | 3,37 | PPP2R1A,YWHAG,YWHAH,ARAF,YWHAB,PPP2R2A,TLN1 |
|  | Proline Biosynthesis II (from Arginine) | 3,18 | PYCR3,OAT |
|  | Arginine Degradation VI (Arginase 2 Pathway) | 3,18 | PYCR3,OAT |
|  | ERK5 Signaling | 2,99 | YWHAG,YWHAH,YWHAE,YWHAB |
|  | Myc Mediated Apoptosis Signaling | 2,89 | YWHAG,YWHAH,YWHAE,YWHAB |
|  | Caveolar-mediated Endocytosis Signaling | 2,87 | RAB5C,FLNA,DNM2,COPG1 |
|  | Citrulline Biosynthesis | 2,80 | OAT,ALDH18A1 |
|  | 14-3-3-mediated Signaling | 2,71 | YWHAG,YWHAH,YWHAE,TUBB6,YWHAB |
|  | Purine Nucleotides De Novo Biosynthesis II | 2,62 | ADSL,IMPDH2 |
|  | Sirtuin Signaling Pathway | 2,43 | PRKDC,ATP5F1C,TIMM44,TIMM50,ACLY,SLC25A5,VDAC2 |
|  | Pyrimidine Ribonucleotides De Novo Biosynthesis | 2,41 | CAD,CTPS1,DDX3X |
|  | ATM Signaling | 2,36 | USP7,PPP2R1A,PPP2R2A,SMC1A |
|  | Superpathway of Citrulline Metabolism | 2,35 | OAT,ALDH18A1 |
|  | Dopamine-DARPP32 Feedback in cAMP Signaling | 2,29 | PPP2R1A,CDK5,PPP2R2A,ATP2A2,PPP3CA |
|  | IGF-1 Signaling | 2,24 | YWHAG,YWHAH,YWHAE,YWHAB |
|  | Protein Kinase A Signaling | 2,23 | YWHAG,YWHAH,YWHAE,YWHAB,FLNA,RACK1,TIMM50,PPP3CA |
|  | Role of CHK Proteins in Cell Cycle Checkpoint Control | 2,17 | PCNA,PPP2R1A,PPP2R2A |
|  | Acetyl-CoA Biosynthesis III (from Citrate) | 2,17 | ACLY |
|  | Huntington's Disease Signaling | 2,15 | ATP5F1C,CDK5,CLTC,RACK1,HSPA9,DNM2 |
|  | Induction of Apoptosis by HIV1 | 2,09 | SLC25A13,SLC25A3,SLC25A5 |
|  | Mitotic Roles of Polo-Like Kinase | 2,00 | PPP2R1A,PPP2R2A,SMC1A |
|  | ILK Signaling | 1,96 | PPP2R1A,CFL1,PPP2R2A,FLNA,IRS4 |
|  | Cysteine Biosynthesis III (mammalia) | 1,95 | CBS/CBSL,PRMT5 |
|  | Remodeling of Epithelial Adherens Junctions | 1,94 | RAB5C,TUBB6,DNM2 |
|  | Clathrin-mediated Endocytosis Signaling | 1,88 | RAB5C,CLTC,USP9X,PPP3CA,DNM2 |
|  | Palmitate Biosynthesis I (Animals) | 1,87 | FASN |
|  | Uridine-5'-phosphate Biosynthesis | 1,87 | CAD |
|  | Fatty Acid Biosynthesis Initiation II | 1,87 | FASN |
|  | Cysteine Biosynthesis/Homocysteine Degradation | 1,87 | CBS/CBSL |
|  | Cardiac β-adrenergic Signaling | 1,81 | PPP2R1A,PPP2R2A,RACK1,ATP2A2 |
|  | Inosine-5'-phosphate Biosynthesis II | 1,70 | ADSL |
|  | Biotin-carboxyl Carrier Protein Assembly | 1,70 | ACACA |
|  | Cell Cycle Regulation by BTG Family Proteins | 1,59 | PPP2R1A,PPP2R2A |
|  | Superpathway of Methionine Degradation | 1,59 | CBS/CBSL,PRMT5 |
|  | Arginine Degradation I (Arginase Pathway) | 1,57 | OAT |
|  | Salvage Pathways of Pyrimidine Ribonucleotides | 1,55 | PYCR3,CDK5,ARAF |
|  | Mitochondrial Dysfunction | 1,54 | ATP5F1C,ATP5PO,AIFM1,VDAC2 |
|  | CTLA4 Signaling in Cytotoxic T Lymphocytes | 1,53 | PPP2R1A,PPP2R2A,CLTC |
|  | CDK5 Signaling | 1,53 | PPP2R1A,CDK5,PPP2R2A |
|  | Tetrahydrofolate Salvage from 5,10-methenyltetrahydrofolate | 1,48 | MTHFD1L |
|  | Folate Polyglutamylation | 1,48 | MTHFD1L |
|  | Protein Ubiquitination Pathway | 1,47 | USP7,USP9X,HSPA9,DNAJA1,PSMC3 |
|  | Stearate Biosynthesis I (Animals) | 1,45 | FASN,SLC27A4 |
|  | Pyrimidine Ribonucleotides Interconversion | 1,43 | CTPS1,DDX3X |
|  | Virus Entry via Endocytic Pathways | 1,42 | FLNA,CLTC,DNM2 |
|  | p53 Signaling | 1,41 | PRKDC,PCNA,COQ8A |
|  | Arginine Biosynthesis IV | 1,40 | OAT |
|  | Acetyl-CoA Biosynthesis I (Pyruvate Dehydrogenase Complex) | 1,33 | PDHB |
|  | 3-phosphoinositide Biosynthesis | 1,32 | ATP1A1,PGAM5,PI4KA,PPP3CA |
| NUCLEAR EXTRACTS | Mitotic Roles of Polo-Like Kinase | 2,66 | SMC3,SMC1A |
|  | ATM Signaling | 2,33 | SMC3,SMC1A |
|  | Estrogen Receptor Signaling | 2,10 | PRKDC,PHB2 |
|  | 14-3-3-mediated Signaling | 2,08 | VIM,TUBA1C |
|  | DNA Double-Strand Break Repair by Non-Homologous End Joining | 1,84 | PRKDC |
|  | Granzyme B Signaling | 1,78 | PRKDC |
|  | RAN Signaling | 1,75 | RANBP2 |
|  | ILK Signaling | 1,75 | IRS4,VIM |
|  | Tumoricidal Function of Hepatic Natural Killer Cells | 1,60 | AIFM1 |
|  | Role of JAK2 in Hormone-like Cytokine Signaling | 1,46 | IRS4 |
|  | Sirtuin Signaling Pathway | 1,43 | PRKDC,TUBA1C |
|  | Pyrimidine Ribonucleotides Interconversion | 1,34 | DDX3X |
|  | Pyrimidine Ribonucleotides De Novo Biosynthesis | 1,32 | DDX3X |

Supplementary Table 7. List of antibodies for the validation of selected ANKRD55 interacting partners. Abbreviations: WB, western blot; IF, immunofluorescence; IP immunoprecipitation.

| Antibody | Catalog Number | Host | Applications |
| --- | --- | --- | --- |
| ANKRD55 | HPA051049  Sigma-Aldrich | Rabbit polyclonal | WB, IF |
| TUBB | 66240-1-Ig Proteintech | Mouse monoclonal CloneNo.: 1D4A4 | WB, IF, IP |
| VIM | 60330-1-Ig Proteintech | Mouse monoclonal CloneNo.: 3H9D1 | WB, IF |
| 14-3-3 (pan) | 8312  Cell Signaling | Rabbit polyclonal | WB |
| 14-3-3  (epsilon) | 11648-2-AP  Proteintech | Rabbit polyclonal | IP |
| 14-3-3 | 66061-1-Ig  Proteintech | Mouse monoclonal  CloneNo.: 2E10A3 | IF |
| RPS3 | 66046-1-Ig Proteintech | Mouse monoclonal CloneNo.: 2G7H4 | WB, IF |
| RPS3 | 11990-1-AP  Proteintech | Rabbit polyclonal | WB, IP |
| CLTC | 66487-1-Ig Proteintech | Mouse monoclonal CloneNo.: 1B3D7 | WB |
| SMC1A | 21695-1-AP Proteintech | Rabbit polyclonal | WB |
| SMC3 | 5696  Cell Signaling | Rabbit monoclonal | WB |
| PRKDC | NBP2-22128SS Novus Biologicals | Mouse monoclonal | WB |
| HIF1AN | NB100-428 Novus Biologicals | Rabbit polyclonal | WB |
| IgG  (control) | 30000-0-AP  Proteintech | Rabbit polyclonal | IP |
| Mouse IgG | B900620  Proteintech | Mouse polyclonal | IP |
| FLAG | 20543-1-AP Proteintech | Rabbit polyclonal | WB, IF |

Supplementary Table 8. List of identified ANKRD55-interacting proteins in protein-protein databases

| Gene Symbol | Accession | Protein name | Resources | Specie |
| --- | --- | --- | --- | --- |
| FHL2 | Q14192 | Four and a half LIM domains protein 2 | HuRI (HI-III) | ***Homo sapiens*** |
| HIF1AN | Q9NWT6 | Hypoxia-inducible factor 1-alpha inhibitor | BioGrid |  |
| IFT52 | Q9Y366 | Intraflagellar transport protein 52 homolog | BioGrid |  |
| ANKS1A | Q49AR9 | Ankyrin repeat and SAM domain-containing protein 1A | HuRI (HI-III) |  |
| PTK6 | Q13882 | Protein-tyrosine kinase 6 | HuRI (HI-III) |  |
| HSF2BP | O75031 | Heat shock factor 2-binding protein | HuRI (HI-III) |  |
| BANP | Q8N9N5 | Protein BANP | HuRI (HI-III) |  |
| CABP5 | Q9NP86 | Calcium-binding protein 5 | HuRI (HI-III) |  |
| CLIC3 | O95833 | Chloride intracellular channel protein 3 | HuRI (HI-III) |  |
| ABI2 | Q9NYB9 | Abl interactor 2 | HuRI (HI-III) |  |
| BOLA2 | A0A087WZT3 | BolA-like protein 2 | HuRI (HI-III) |  |
| HGS | O14964 | Hepatocyte growth factor-regulated tyrosine kinase substrate | HuRI (HI-III) |  |
| FRS3 | O43559 | Fibroblast growth factor receptor substrate 3 | HuRI (HI-III) |  |
| NCK2 | O43639 | Cytoplasmic protein NCK2 | HuRI (HI-III) |  |
| TRIM23 | P36406 | E3 ubiquitin-protein ligase TRIM23 | HuRI (HI-III) |  |
| TRIP6 | Q15654 | Thyroid receptor-interacting protein 6 | BioGrid, HuRI (HI-III, unpublished GS test space) |  |
| NOXA1 | [Q86UR1](http://www.uniprot.org/uniprot/Q86UR1) | NADPH oxidase activator 1 | HuRI (HI-III) |  |
| TTC30A | [Q86WT1](http://www.uniprot.org/uniprot/Q86WT1) | Tetratricopeptide repeat protein 30A | BioGrid |  |
| CFAP206 | [Q8IYR0](http://www.uniprot.org/uniprot/Q8IYR0) | Cilia- and flagella-associated protein 206 | HuRI (HI-III) |  |
| TTC30B | [Q8N4P2](http://www.uniprot.org/uniprot/Q8N4P2) | Tetratricopeptide repeat protein 30B | BioGrid |  |
| TEKT4 | [Q8WW24](http://www.uniprot.org/uniprot/Q8WW24) | Tektin-4 | HuRI (HI-III) |  |
| ZMYND19 | [Q96E35](http://www.uniprot.org/uniprot/Q96E35) | Zinc finger MYND domain-containing protein 19 | HuRI (HI-III) |  |
| IFT74 | [Q96LB3](http://www.uniprot.org/uniprot/Q96LB3) | Intraflagellar transport protein 74 homolog | BioGrid |  |
| PFDN5 | [Q99471](http://www.uniprot.org/uniprot/Q99471) | Prefoldin subunit 5 | HuRI (HI-III) |  |
| BACH2 | [Q9BYV9](http://www.uniprot.org/uniprot/Q9BYV9) | Transcription regulator protein BACH2 | HuRI (HI-III) |  |
| IFT46 | Q9NQC8 | Intraflagellar transport protein 46 homolog | BioGrid |  |
| ZSCAN1 | Q8NBB4 | Zinc finger and SCAN domain-containing protein 1 | BioGrid |  |
| YWHAZ | [P63104](http://www.uniprot.org/uniprot/P63104) | 14-3-3 protein zeta/delta | BioGrid, |  |
| MIB1 | [Q86YT6](http://www.uniprot.org/uniprot/Q86YT6) | E3 ubiquitin-protein ligase MIB1 | BioGrid |  |
| YWHAG | [P61981](http://www.uniprot.org/uniprot/P61981) | 14-3-3 protein gamma | BioGrid |  |
| RABGEF1 | Q9UJ41 | Rab5 GDP/GTP exchange factor | HuRI (HI-III) |  |
| LEF1 | Q9UJU2 | Lymphoid enhancer-binding factor 1 | HuRI (HI-III, unpublished GS test space) |  |
| SUFU | Q9UMX1 | Suppressor of fused homolog | HuRI (HI-III, unpublished GS test space) |  |
| CARD9 | Q9H257 | Caspase recruitment domain-containing protein 9 | HuRI (HI-III, unpublished GS test space) |  |
| DEUP1 | Q05D60 | Deuterosome assembly protein 1 | HuRI (HI-III, unpublished GS test space) |  |
| LRP2BP | P98164 | Low-density lipoprotein receptor-related protein 2 | HuRI (HI-III, unpublished GS test space) |  |
| SF3A3 | Q12874 | Splicing factor 3A subunit 3 | HuRI (HI-III, unpublished GS test space) |  |
| TCAP | O15273 | Telethonin | HuRI (HI-III, unpublished GS test space) |  |
| FANCL | Q9NW38 | E3 ubiquitin-protein ligase FANCL | HuRI (HI-III, unpublished GS test space) |  |
| CIAO1 | O76071 | Probable cytosolic iron-sulfur protein assembly protein CIAO1 | HuRI (HI-III, unpublished GS test space) |  |
| YWHAE | P62258 | 14-3-3 protein epsilon | BioGrid |  |
| YWHAQ | P27348 | 14-3-3 protein theta | BioGrid |  |
| RPS3 | P23396 | 40S ribosomal protein S3 | STRING |  |
| CNOT6L | Q96LI5 | CCR4-NOT transcription complex subunit 6-like | STRING |  |
| LRKK2 | Q5S007 | Leucine-rich repeat serine/threonine-protein kinase 2 | STRING |  |
| NNT | Q13423 | NAD(P) transhydrogenase, mitochondrial | STRING |  |
| ASH1L | Q9NR48 | Histone-lysine N-methyltransferase ASH1L | STRING |  |
| PHLPP1 | O60346 | PH domain leucine-rich repeat-containing protein phosphatase 1 | STRING |  |
| PHLPP2 | Q6ZVD8 | PH domain leucine-rich repeat-containing protein phosphatase 2 | STRING |  |
| LRGUK | Q96M69 | Leucine-rich repeat and guanylate kinase domain-containing protein | STRING |  |
| GART | P22102 | Trifunctional purine biosynthetic protein adenosine-3 | STRING |  |
| ACLY | P53396 | ATP-citrate synthase | STRING |  |
| Nnt | Q61941 | NAD(P) transhydrogenase, mitochondrial | STRING | ***Mus musculus*** |
| Rps3 | P62908 | 40S ribosomal protein S3 | STRING |  |
| Gart | Q64737 | Trifunctional purine biosynthetic protein adenosine-3 | STRING |  |
| Phlpp2 | Q8BXA7 | PH domain leucine-rich repeat-containing protein phosphatase 2 | STRING |  |
| Nos | Q9Z0J4 | Nitric oxide synthase, brain | STRING |  |
| Hsp90aa1 | P07901 | Heat shock protein HSP 90-alpha | STRING |  |
| Hsp90ab1 | P11499 | Heat shock protein HSP 90-beta | STRING |  |
| Phlpp1 | Q8CHE4 | PH domain leucine-rich repeat-containing protein phosphatase 1 | STRING |  |
| Lrrk1 | Q3UHC2 | Leucine-rich repeat serine/threonine-protein kinase 1 | STRING |  |
| Lrrk2 | Q5S006 | Leucine-rich repeat serine/threonine-protein kinase 2 | STRING |  |

Supplementary Table 9. List of potential 14-3-3 binding sites in ANKRD55. Highly scored phosphopeptides by one (green), two (blue) or three (red) methods are shown. Abbreviations: ANN, Artificial Neural Network (cut-off = 0.55); PSSM Position-Specific Scoring Matrix (cut-off = 0.80); SVM, Support Vector Machine (cut-off = 0.25). Consensus - Average of the scores provided by the three methods (cut-off = 0.50). Highly scored by one (green), two (blue) or three (red) methods.

| 14-3-3 binding site | Predicted phosphopeptides | ANN | PSSM | SVM | Consensus |
| --- | --- | --- | --- | --- | --- |
| 36 | MVYQAAsNGDV | 0.577 | 0.239 | -0.278 | 0.18 |
| 59 | ILECCDsEGCT | 0.607 | 0.107 | -0.203 | 0.17 |
| 112 | WLEGCVsLLRN | 0.609 | 0.064 | -0.058 | 0.205 |
| 323 | LSQESRtEPTR | 0.609 | 0.691 | 0.236 | 0.512 |
| 436 | PPIRTQsLPPI | 0.917 | 1.494 | 1.525 | 1.312 |
| 455 | ASHRATsHAGL | 0.569 | 0.733 | 0.228 | 0.51 |
| 475 | RSQKSRsEQDL | 0.715 | 0.46 | 0.213 | 0.463 |
| 597 | PSQRRHsTAAE | 0.846 | 1.106 | 0.778 | 0.91 |
| 598 | SQRRHStAAEE | 0.635 | 0.964 | 0.134 | 0.577 |
